# Ritonavir and xk263 Binding-Unbinding with HIV-1 Protease: Pathways, Energy and Comparison

**DOI:** 10.3390/life12010116

**Published:** 2022-01-13

**Authors:** Jianan Sun, Mark Anthony V. Raymundo, Chia-En A. Chang

**Affiliations:** 1 Environmental Toxicology Graduate Program, University of California, Riverside, CA 92521, USA; jianan.sun@email.ucr.edu; 2 Department of Chemistry, University of California, Riverside, CA 92521, USA; mraymundo@palomar.edu

**Keywords:** energy barrier, enhanced sampling, residence time, ligand–protein recognition, drug design

## Abstract

Understanding non-covalent biomolecular recognition, which includes drug–protein bound states and their binding/unbinding processes, is of fundamental importance in chemistry, biology, and medicine. Fully revealing the factors that govern the binding/unbinding processes can further assist in designing drugs with desired binding kinetics. HIV protease (HIVp) plays an integral role in the HIV life cycle, so it is a prime target for drug therapy. HIVp has flexible flaps, and the binding pocket can be accessible by a ligand via various pathways. Comparing ligand association and dissociation pathways can help elucidate the ligand–protein interactions such as key residues directly involved in the interaction or specific protein conformations that determine the binding of a ligand under certain pathway(s). Here, we investigated the ligand unbinding process for a slow binder, ritonavir, and a fast binder, xk263, by using unbiased all-atom accelerated molecular dynamics (aMD) simulation with a re-seeding approach and an explicit solvent model. Using ritonavir-HIVp and xk263-HIVp ligand–protein systems as cases, we sampled multiple unbinding pathways for each ligand and observed that the two ligands preferred the same unbinding route. However, ritonavir required a greater HIVp motion to dissociate as compared with xk263, which can leave the binding pocket with little conformational change of HIVp. We also observed that ritonavir unbinding pathways involved residues which are associated with drug resistance and are distal from catalytic site. Analyzing HIVp conformations sampled during both ligand–protein binding and unbinding processes revealed significantly more overlapping HIVp conformations for ritonavir-HIVp rather than xk263-HIVp. However, many HIVp conformations are unique in xk263-HIVp unbinding processes. The findings are consistent with previous findings that xk263 prefers an induced-fit model for binding and unbinding, whereas ritonavir favors a conformation selection model. This study deepens our understanding of the dynamic process of ligand unbinding and provides insights into ligand–protein recognition mechanisms and drug discovery.

## 1. Introduction

HIV type 1 (HIV-1) garnered enormous attention in the 1970s because it can attack CD4 cells and weaken the immune system, eventually causing acquired immunodeficiency syndrome (AIDS) if not suppressed in vivo. HIV protease (HIVp) is one of the essential proteins in the HIV life cycle, responsible for cleaving premature protein and producing fully functional enzymes [1,2,3]. Protease inhibitors (PIs) can disrupt HIVp function and stop HIV replication via competitive inhibition [4,5]. However, PI-selected mutations decrease the HIVp susceptibility to inhibitors and induce cross-resistance among PIs [6,7,8].

HIVp is a good model system to study ligand–protein binding/unbinding because HIVp is a homodimer with well-defined regions. Flap regions in HIVp must open in order to bring the peptide into the binding pocket [9,10]. Then the flaps close. Four residues, Asp25, Asp124, Ile50 and Ile149, are essential for holding the peptide in place, with Asp25 and Asp124 catalyzing proteolysis [11]. Such well-defined structural behavior and ligand–protein interaction features are informative in investigating protein conformational change during ligand–protein binding/unbinding.

Non-covalently binding drugs can reversibly bind and unbind from their protein targets. Many drugs forfeit their pharmacological effect once they leave the target site. In vivo and in silico studies suggest that pharmacological activity depends on the drug lifetime [12,13,14]. Binding (k_on_) and unbinding rate constants (k_off_) are two factors used to describe the kinetic process of the drug–protein interaction. Ideally, we want a drug with large k_on_ (faster binding) and small k_off_ (slower leaving). Residence time, the reciprocal of the unbinding rate constant, describes the lifetime of a ligand in the bound state with its target protein. Developing drugs with long residence time can enhance the pharmacological effect. All-atom simulations of ligand–protein dissociation can provide mechanistic information at the atomistic level [15,16]. Investigating the interactions between the drug and protein during unbinding may provide insights into drug design and altering drug residence time.

Several recent studies have focused on the binding kinetics of drug–HIVp systems [17,18,19,20]. Enhanced sampling methods, such as steered molecular dynamics (SMD), metadynamics, and accelerated MD (aMD), were used to sample ligands binding or unbinding from HIVp [21,22,23,24]. Binding kinetics explain the dynamic processes of ligand binding/unbinding, not merely the difference between the bound state and free state. Two binding mechanisms, induced-fit and conformation-selection, have been proven in experimental and computational studies [25,26,27]. Different ligands show distinct binding kinetics [28,29]. For example, ritonavir requires HIVp conformational change to bind, whereas xk263 can bind to HIVp without obvious protein motions [27]. The free energy profile was constructed computationally to reveal relative free energies between various intermediate, free and ligand bound states for ligand–HIVp binding or unbinding [30,31,32]. However, because of the complex dynamic nature of HIVp when binding to ligands with diverse properties, we still lack comprehensive studies explaining the possible unbinding pathway(s) and protein conformational change during ligand unbinding from HIVp.

Here, we studied processes of unbinding of a pair of ligands, ritonavir and xk263, from HIV-1 protease. Investigation into how differently these two ligands interact with HIVp provides insights into factors that affect the unbinding processes. We classify unbinding trajectories and discuss ligand unbinding pathways, intermolecular H-bond networks, and HIVp conformations during the dissociation processes. Root-mean-square deviation (RMSD)-based analysis was used to identify overlapping HIVp conformations between xk263 or ritonavir binding and unbinding. We also discuss the ligand unbinding mechanisms and compare these with ligand–protein binding models.

## 2. Materials and Methods

### 2.1. Target Molecular Systems

**Figure 1:**
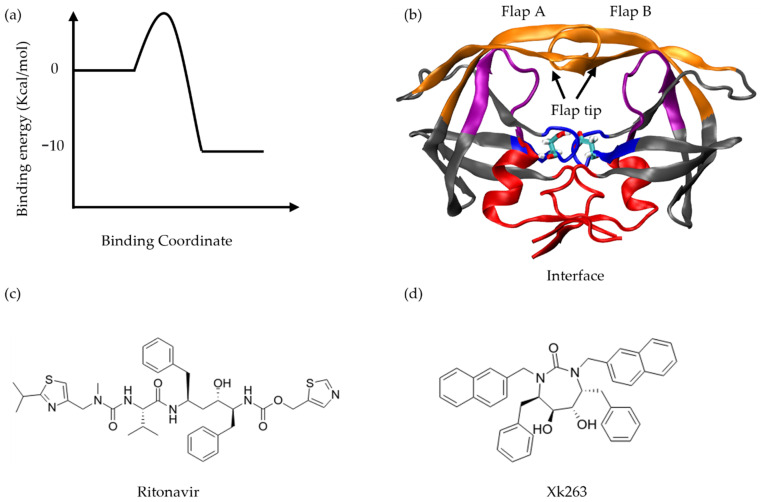
(**a**) Free energy difference between ligand–protein unbound state and bound state. (**b**) HIV protease (HIVp) structure with color-coded regions: flap (orange, flap a: residues 42–60, flap b: residues 141–159, flap tips: residues 49–52 and 148–151), loop (pink, residues: 75–85 and 174–184), catalytic triads (blue, residues: 23–29 and 122–128) and dimer interface (red, residues: 1–9, 86–99, 100–108 and 185–198). Catalytic residues, Asp 25 and Asp 124, are shown in licorice. Two-dimensional structure of (**c**) ritonavir and (**d**) xk263.

We selected Protein Data Bank entry 1HXW and 1HVR to study ritonavir and xk263 unbinding from HIVp, respectively (Figure 1b–d) [33,34]. 1HXW contains a ritonavir–HIVp crystal structure in the bound state, with a single protonation state applied for Asp25. 1HVR contains an xk263–HIVp crystal structure in the bound state, with a double protonation state for Asp25/124. We applied distinct protonation states for the two systems based on the results of the lowest interaction energy between the ligand and four different protonation states of HIVp [35].

### 2.2. MD Simulations

We performed 100-ns conventional MD (cMD) simulation for ritonavir–HIVp and xk263–HIVp followed by aMD simulations of 25 seeds for each system with a re-seeding approach, as described below.

The Amber18 package with GPU implementation was used for unbiased all-atom MD simulations [36]. The Amber FF99SB and General Amber Force Field (GAFF) were used for HIV protease and ligands, respectively [37]. VCharge was used to assign partial charges for the ligand atoms for better charge accuracy of resonance structures of phenyl groups in ritonavir and phenyl and naphthalenyl groups in xk263 [38]. Five chloride ions and six chloride ions were placed to maintain a neutral system for ritonavir–HIVp and xk263–HIVp, respectively. Minimization of hydrogen atoms, the side chains and the entire system was applied for 1000, 1000 and 3000 steps, respectively. The system was solvated in a rectangular TIP3P water box and the edge of the box was at least 10 Å away from the solutes. The system went through 1000 steps of water minimization and 5000 steps of whole-system minimization to correct any inconsistencies. Then the system was slowly heated to 50, 100, 150, 200, 250 K for 200 ps at each temperature and 9 ns at 300 K. Then, 100-ns cMD was performed in an isothermic−isopressure (NPT) ensemble to ensure that the system reached equilibrium. Langevin Thermostat along with a damping constant of 2 ps^−1^ was used to maintain a temperature of 310 K. Particle mesh Eward (PME) was used to compute the long-range electrostatic interactions > 10 Å [39]. Frames were saved every 10 ps with a time step of 2 fs. Finally, the SHAKE algorithm was used to constrain the covalent bonds involving hydrogen atoms [40].

### 2.3. Accelerated MD (aMD) Simulation

Biomacromolecules can have high energy barriers when a molecule is moving around the energy surface, which prevents efficient conformation sampling in cMD. Therefore, we used the final frame from the cMD run as the initial conformation for aMD simulations with a different starting velocity for each seed. AMD enhances the conformational sampling by adding a continuous non-negative bias or “boost” potential ∆V(r) to the potential energy V(r) whenever the potential energy takes values below some predetermined threshold *E* [41]. A bias may also be applied to specific components of the potential energy, for example, the dihedral energy $V_{D}\left(r\right)$. Energy boosts to both the total potential energy and the dihedral energy are applied here. The overall bias potential is given by

(1) $$∆V\left(r\right)=\frac{{\left(E_{P}-V\left(r\right)\right)}^{2}}{\alpha P+\left(E_{P}-V\left(r\right)\right)}+\frac{{\left(E_{D}-V_{D}\left(r\right)\right)}^{2}}{\alpha D+\left(E_{D}-V_{D}\left(r\right)\right)}$$

where *E_p_* and *E_D_* are the average total potential energy threshold and average dihedral energy threshold based on cMD simulation. αP and αD are boost factors for the total potential and dihedral potential, respectively. The actual values of *E_p_*, *E_D_*, αP and αD are listed in supporting information (Table S1). By increasing the value of the tuning parameter α, we can reduce the energy boost, ∆V(r), thus allowing the conformational change of protein to evolve faster than in cMD yet maintain the protein’s secondary structure. Protein backbone dihedral angle change directly leads to protein conformational change. Hence, we applied both dihedral and total potential energy boost to our model systems. AMD input parameters can be found in Supporting Information.

### 2.4. Re-Seeding Approach

Ritonavir and xk263 are both tight binders to HIVp. To efficiently explore their unbinding pathways, we applied a re-seeding approach under aMD simulation. Re-seeding refers to generating multiple MD simulation production runs from the same initial ligand–protein conformation but with different atom velocities. The last frames from cMD simulations of ritonavir–HIVp and xk263–HIVp were used to provide an initial conformation for 25 ritonavir-HIVp first re-seedings and 25 xk263-HIVp first re-seedings. Each first re-seeding is a 400-ns-long aMD simulation. Five 25-ns-long second re-seedings were applied with the last frame of the first re-seeding as an initial conformation if unbinding did not occur in the 400-ns aMD simulation. The third re-seeding was performed to generate five 25-ns-long aMD simulations using the frame from the second re-seeding in which the ligand reached the largest RMSD as compared with the fully relaxed ligand position in the bound state. The following re-seedings were performed with the same method as generating third-layer re-seeding. If the selected initial frame contained a ligand–HIVp conformation that presented a strong tendency to dissociate, up to twenty 25-ns-long aMD simulations would be generated from such frame. A detailed example of the re-seeding approach can be found in Supplementary Information Figure S1. Re-seeding was repeated until successful ligand dissociation, after the 17th re-seeding, or protein unfolding/distortion. Table S2 lists re-seeding attempts for ritonavir and xk263 dissociations.

### 2.5. Hydrogen Bond Analysis

Each hydrogen bond between the ligand and protein may contribute up to a few kcal/mol to interaction energy [42]. To hydrolyze the peptide bond in premature protein, Asp25/124 establishes hydrogen bonding with the peptide backbone in the substrate, whereas Ile50/149 stabilizes the substrate with a water bridge [35]. To better understand which residues may form H-bonds with ritonavir/xk263 during ligand dissociation, we analyzed and plotted H-bond versus time by using the CPPTRAJ program [36,43]. The criteria for H-bonding are (1) the distance between donor D and acceptor A < 3 Å, and (2) the D-H-A angle, where H is the shared hydrogen, at least 150°.

### 2.6. MM/PBSA Interaction Energy

To investigate the interaction energy between the ligand and HIVp, we used the molecular mechanics Poisson-Boltzmann surface area (MM/PBSA) method [44]. Interaction energy, ΔE, is calculated by

(2) $$∆E=E_{\mathrm{PL}}-E_{P}-E_{L}$$

where $E_{\mathrm{PL}},{\;E}_{P}$ and $E_{L}$ represent enthalpy of the ligand–protein complex, enthalpy of protein and enthalpy of ligand, respectively.

(3) $$E_{\mathrm{MM}/\mathrm{PBSA}}={\;E}_{\mathrm{gas}}+{\;G}_{\mathrm{solvation}}$$

$E_{\mathrm{gas}}$ is gas-phase energy calculated from the molecular mechanical force field, $G_{\mathrm{solvation}}$ is the solvation energy calculated by solving the Poisson-Boltzmann equation. $E_{\mathrm{MM}/\mathrm{PBSA}}$ can be further decomposed as

(4) $$E_{\mathrm{MM}/\mathrm{PBSA}}=E_{\mathrm{bond}}+E_{\mathrm{angle}}+E_{\mathrm{torsion}}+E_{\mathrm{vdW}}+E_{\mathrm{elect}}+\left(G_{\mathrm{pb}}+G_{\mathrm{np}}\right)$$

where $E_{\mathrm{bond}}$ is bonded energy, $E_{\mathrm{angle}}$ is angle energy,${\;E}_{\mathrm{torsion}}$ is dihedral energy, $E_{\mathrm{vdW}}$ is van der Waals (vdW) energy, $E_{\mathrm{elect}}$ is electrostatic energy, $G_{\mathrm{pb}}$ is polar solvation energy, and $G_{\mathrm{np}}$ is non-polar solvation energy. The bonded terms were canceled in interaction energy calculations because $E_{\mathrm{PL}},{\;E}_{P}$ and $E_{L}$ used the same ligand–protein conformation. We can rewrite

(5) $$∆E_{\mathrm{MM}/\mathrm{PBSA}}=∆E_{\mathrm{vdW}}+∆E_{\mathrm{elect}}+∆G_{\mathrm{pb}}+∆G_{\mathrm{np}}$$

(6) $$=∆E_{\mathrm{polar}}+∆E_{\mathrm{non}-\mathrm{polar}}$$

where $∆E_{\mathrm{polar}}$ is polar interaction energy ($∆E_{\mathrm{elect}}+∆G_{\mathrm{pb}}$) and $∆E_{\mathrm{non}-\mathrm{polar}}$ non-polar interaction energy ($∆E_{\mathrm{vdW}}+∆G_{\mathrm{np}}$). Entropy contributions are neglected in MM/PBSA calculations. Polar, non-polar and total interaction energies are calculated with a 1-ns interval. Energies are averaged using three computed energy values, 1 ns before and after a reference frame (average 3 ns). The average energy at 0 ns is the mean of 0 ns and 1 ns, and the average energy at the last timestep is the average of the last and second last timestep.

### 2.7. RMSD-Based Dissociation-Association Trajectory Comparison

If the backbone RMSD of 2 HIVp conformations are within 2.5 Å, we define them as similar conformations or overlapping conformations. Under such criteria, we iteratively compared HIVp conformations in dissociation trajectories with association trajectories. Because pathway A is the most popular dissociation path for both ligands, we selected one ritonavir dissociation trajectory and one xk263 dissociation trajectory under pathway A that are discussed in the following context. We selected eight ritonavir association trajectories and six xk263 association trajectories from previous work [27]. Frames were saved every 1 ps in our original trajectories, and we re-saved a frame every 0.1 ns for each trajectory. Frames from the representative trajectory from pathway A were re-saved every 1 ns as reference structures (every 10 frame) to ensure that the frame count figure was succinct.

Using regions of HIVp from the crystal structure (i.e., regions containing residues 1 to 41, 61 to 130, and 160 to 198, flap regions excluded) from PDB entry 1HXW as a reference frame, we aligned all ritonavir association/dissociation trajectories to clearly observe flap motions. Using 1-ns interval frames in ritonavir dissociation as reference, we calculated HIVp backbone RMSD for all frames in each of eight association trajectories, then plotted a bar graph, similar frame count versus each reference frame, for visualizing the comparison result. The same analysis was conducted for the xk263–HIVp system using partial 1HVR as a reference frame for alignment.

## 3. Results

Figure 1a illustrates a schematic of non-covalent binding free energy, with two molecules binding and unbinding when a system reaches equilibrium. Although binding and unbinding may share highly similar paths, ligand unbinding may not need to follow the same path as it binds to the protein target. Here, we first classified unbinding pathways for two ligands with different chemical properties, and then compared the association and dissociation processes to further understand the binding and unbinding processes.

**Figure 2:**
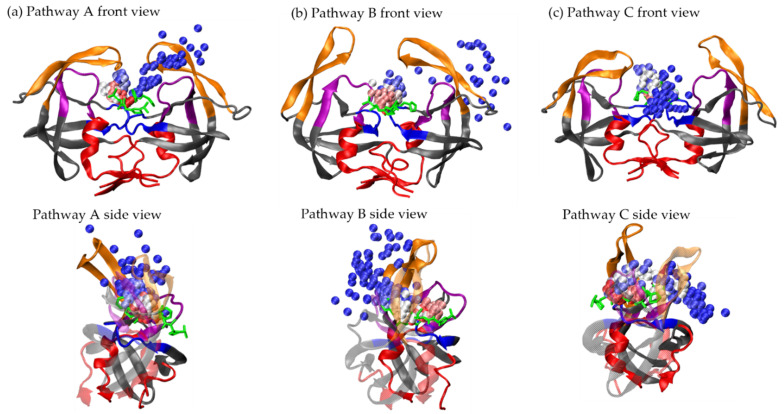
Dissociation pathways of HIVp and ritonavir sampled by accelerated molecular dynamics (aMD). Each bead represents a position of center of mass of ritonavir with 1-ns interval during dissociation, with total simulation time of 248.7 ns, 304.9 ns and 543.1 ns for pathways A (**a**), B (**b**), and C (**c**), respectively. Color beads present center of mass of ritonavir taken from frames in the beginning (red), middle (white) and near the end (blue) of the trajectory. Ritonavir’s initial position is green licorice. Flap region (orange), loop region (purple), catalytic triads (blue) and interface region (red) are colored for better visualization. The HIVp conformation is taken from the final frame in each dissociation trajectory.

**Figure 3:**
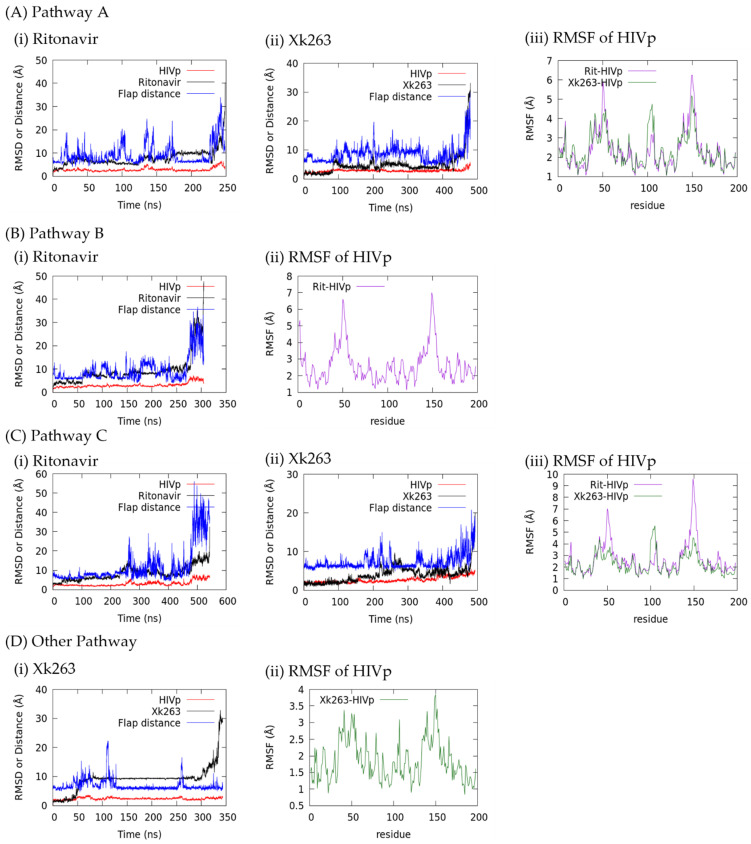
Binding pocket openness, ligand RMSD and HIVp RMSD ((**A**,i and ii), (**B**,i), (**C**,i and ii) and (**D**,i)) as well as HIVp RMSF((**A**,iii), (**B**,ii), (**C**,iii) and (**D**,ii)) under different pathways. We use flap tip distance (αC Ile 50 to αC Ile 149) to describe flap behavior and binding pocket openness. Distance, RMSD and RMSF are in angstroms.

Using the re-seeding approach described in Methods, we obtained 20 and 15 unbinding trajectories for ritonavir and xk263, respectively. We grouped the trajectories into four unbinding routes based on where a ligand diffuses after leaving the binding pocket of HIVp—pathway A: unbinding between the flap and loop region; pathway B: surface diffusion through the flap region; pathway C: surface diffusion through the interface region; and others (Table 1 and Figure 2). Both ritonavir and xk263 preferred to dissociate from HIVp under pathway A because in 11 of 20 dissociation trajectories and 8 of 15 dissociation trajectories, ritonavir and xk263 followed the flap/loop unbinding pathway, respectively. We reported binding pocket openness and flap motions using the distance between Cα of Ile 50-Ile 149 (flap tip). Figure 3 also shows ligand RMSD (ritonavir/xk263) and HIVp RMSD and RMSF for easy comparison between molecular motions, pathways and different ligands. We performed H-bond analysis and intermolecular energy calculation by using MM/PBSA during ligand unbinding [43]. We also examined conformations from our dissociation pathways with reported association pathways to reveal popular HIVp conformations sampled during binding/unbinding.

### 3.1. Ligand Unbinding Pathways

Here, we discuss the pathways in detail to understand the similarities and differences between ritonavir and xk263 during unbinding.

#### 3.1.1. Pathway A: Dissociation between Flap/Loop Region

**Figure 4:**
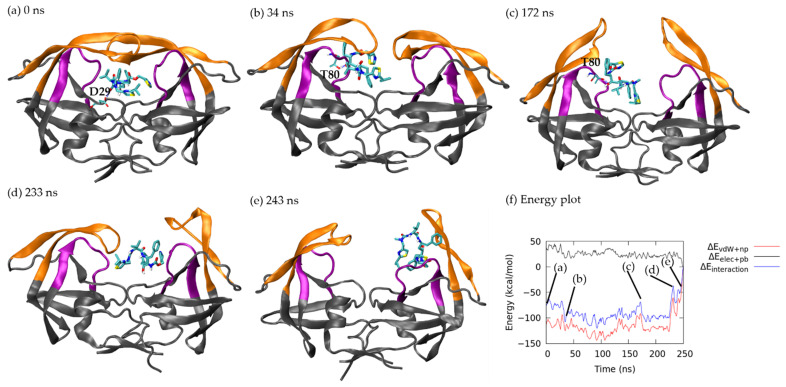
Ritonavir dissociation under pathway A. Flap and loop are in orange and purple, respectively. Ritonavir is in licorice. Hydrogen bond (H-bond) is shown in dashed blue line with the corresponding residue labelled. (**a**) 0 ns. (**b**) 34 ns. (**c**) 172 ns. (**d**) 233 ns. (**e**) 243 ns. (**f**) Non-polar interaction energy (red), polar interaction energy (black) and total interaction energy (blue) between ritonavir and HIVp during unbinding.

Pathway A is the major unbinding pathway for both ligands because 11 of 20 ritonavir dissociation trajectories and 8 of 15 xk263 dissociation trajectories presented unbinding through the flap/loop region of HIVp (Figure 2a). Flaps opened and closed repeatedly during ligand dissociation because the distance between αC Ile 50 and αC Ile 149 fluctuated constantly. Previous studies showed that free HIVp has flaps that open/closed spontaneously, and the same behavior was preserved when in the ritonavir bound complex [27,45,46]. At the near end of ritonavir or xk263 dissociation, the protein underwent a wide-open handedness, which allowed the ligand to unbind from HIVp. RMSF analysis of HIVp during ligand dissociation revealed that the flap region experienced larger motion with ritonavir than xk263 dissociation (Figure 3(A,iii)). H-bonds between both ligands and the loop region were essential to stabilize ligands during unbinding, and yet a strong H-bond may be a disservice for ritonavir dissociation. As shown in Figure 4b, ritonavir can form an H-bond with Thr 80 in the loop region for a long simulation time (Figure S2), which strengthens the ritonavir-HIVp interaction and might prevent dissociation, whereas the H-bond between xk263 and Thr 80 was rarely observed. However, it took much longer for xk263 to leave the binding pocket because of the strong H-bond network; xk263 must break the H-bond network formed from both the flap tip and catalytic triads to unbind. The H-bond network restriction and the rigidity of xk263 led to smaller ligand motion on unbinding with ~5 Å RMSD from 100 ns to 400 ns as compared to ~8 Å RMSD for ritonavir from 20 ns to 215 ns (Figure 3(A,i,ii)). When we restarted simulations using reseeding strategy, xk263 and ritonavir frequently returned to the binding pocket even when already in the flap/loop region. Only when the ligand passed the gap between the flap/loop, it quickly solvated and left HIVp.

**Figure 5:**
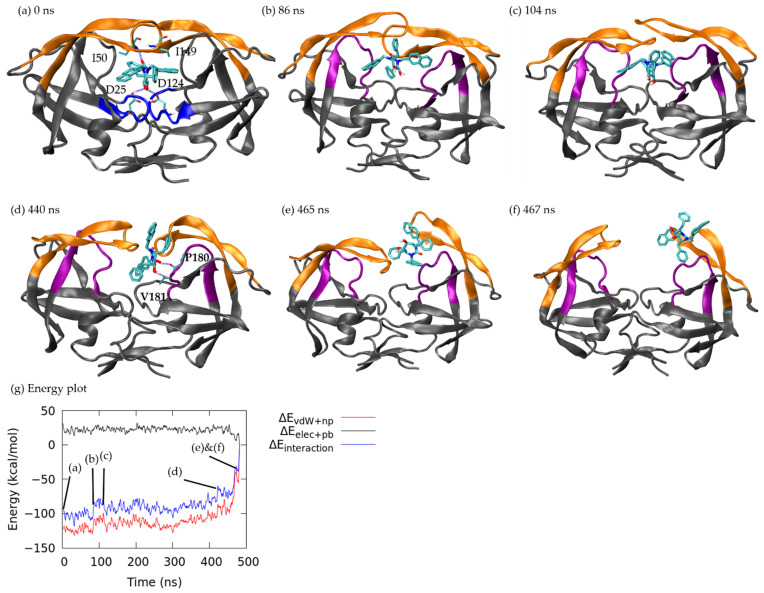
Xk263 dissociation under pathway A. Flap and loop are in orange and purple, respectively; xk263 is in licorice. H-bond is shown in dashed blue line with the corresponding residue labelled. (**a**) 0 ns. (**b**) 86 ns. (**c**) 104 ns. (**d**) 440 ns. (**e**) 465 ns. (**f**) 467 ns. (**g**) Nonpolar interaction energy (red), polar interaction energy (black) and total interaction energy (blue) between xk263 and HIVp during unbinding.

**Figure 6:**
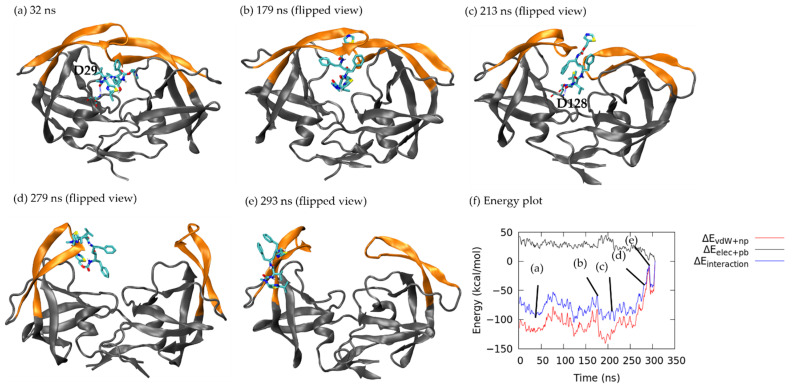
Ritonavir dissociation under pathway B. Flap is in orange. Ritonavir is in licorice. H-bond is shown in dashed blue line with the corresponding residue labelled. (**a**) 31 ns. (**b**) 179 ns. HIVp and ritonavir were flipped along the horizontal in (**c**) 213 ns, (**d**) 279 ns and (**e**) 293 ns for better visualization. (**f**) Non-polar interaction energy (red), polar interaction energy (black) and total interaction energy (blue) between ritonavir and HIVp during unbinding.

**Figure 7:**
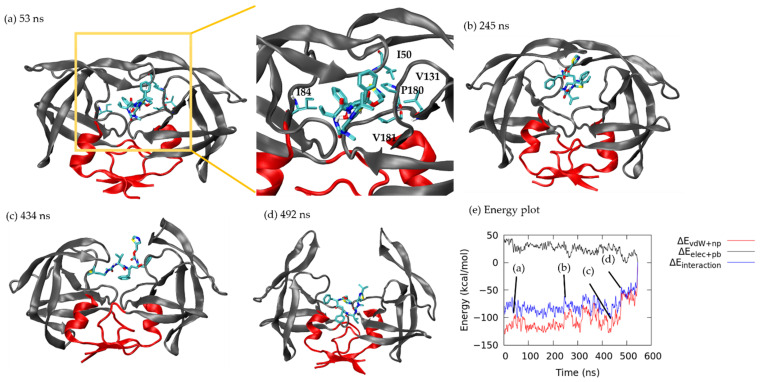
Ritonavir dissociation under pathway C. Interface region is in red. Ritonavir is in licorice. (**a**) 53 ns. (**b**) 245 ns. (**c**) 434 ns. (**d**) 492 ns. (**e**) Non-polar interaction energy (red), polar interaction energy (black) and total interaction energy (blue) between ritonavir and HIVp during unbinding.

**Figure 8:**
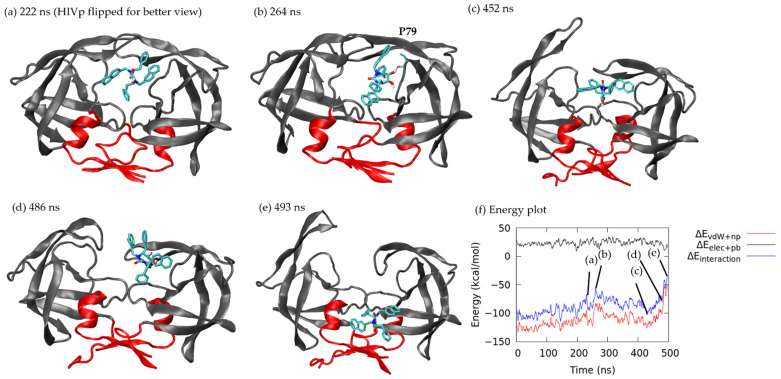
Xk263 dissociation under pathway C. Interface region is in red. Xk263 is in licorice. H-bond is shown in dashed blue line with the corresponding residue labelled. (**a**) 222 ns. (**b**) 264 ns. (**c**) 452 ns. (**d**) 486 ns. (**e**) 493 ns. (**f**) Non-polar interaction energy (red), polar interaction energy (black) and total interaction energy (blue) between xk263 and HIVp during unbinding.

**Figure 9:**
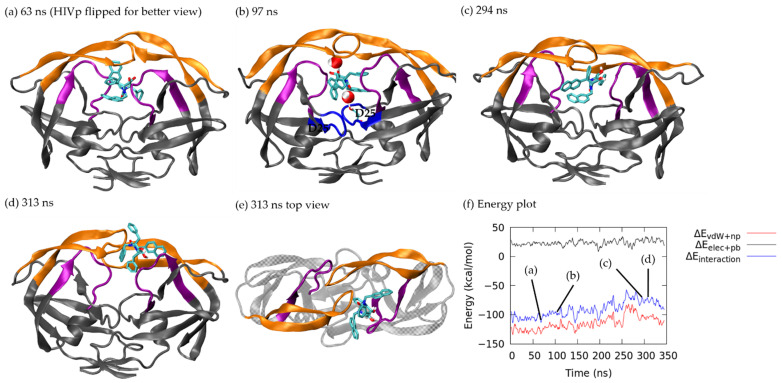
Xk263 dissociation with closed-flap HIVp. Flap, loop and catalytic triads are in orange, purple and blue. Xk263 is in licorice. H-bond is shown in dashed blue line with the corresponding residue labelled. (**a**) 63 ns. (**b**) 97 ns. (**c**) 294 ns. (**d**,**e**) 313 ns. (**f**) Non-polar interaction energy (red), polar interaction energy (black) and total interaction energy (blue) between xk263 and HIVp during unbinding.

Here we computed the interaction energy to further understand the dissociation processes and the intermolecular attractions. Although ritonavir has more H-bond donor/acceptor atoms, the drug does not have stronger polar attraction with HIVp as compared with xk263. The polar interactions consider both electrostatic attraction and PB solvation free energy. Our calculations show that non-polar attraction is the dominant energy term during ligand dissociation for both ritonavir and xk263 (Figure 4c, Figure 5g, Figure 6f, Figure 7e, Figure 8f and Figure 9f).

In the beginning of the aMD run (Figure 4a), the interaction energy between ritonavir and HIVp was −76.11 kcal/mol (non-polar: −113.46 kcal/mol, polar: 37.35 kcal/mol). Notably, we focused on intermolecular interactions. The solute conformational energy and the entropic effects are not included here. As ritonavir rearranged and moved toward one side of the flap/loop region of HIVp, the system reached a local energy minima of −107.48 kcal/mol (non-polar: −124.43 kcal/mol, polar: 16.95 kcal/mol) at 34 ns because of increased contact area and the H-bond formation between hydroxyl groups in Thr 80 and ritonavir (Figure 4b). The H-bond length was 1.67 Å at 34 ns and remained so until 228 ns (Figure S2), and flap A and B opened and closed repeatedly. Ritonavir also formed a H-bond with Asp 29 and Arg 107 from 1 ns to 170 ns, which stabilized ritonavir, and the RMSD of ritonavir was maintained within 8 Å as compared with its bound-state position (Figure S2 and Figure 3a). At 172 ns, HIVp rearranged to a wide-open conformation, which kept the ritonavir–Thr 80 H-bond (1.77 Å) but significantly weakened the non-polar intermolecular interactions, thus resulting in an energy barrier of −68.72 kcal/mol (non-polar: −91.49 kcal/mol, polar: 22.77 kcal/mol) (Figure 4c). As ritonavir migrated from chain A (Figure 4c) to chain B (Figure 4d), an energy barrier of −51.05 kcal/mol (non-polar: −63.16 kcal/mol, polar: 25.62 kcal/mol) occurred because of the reduced contacts between the two molecules. Once ritonavir contacted flap B at 234 ns (Figure 4e), it moved to the gap between the flap/loop region and was temporarily stabilized with interaction energy of −52.76 kcal/mol (non-polar: −70.88 kcal/mol, polar: 18.12 kcal/mol). Then, ritonavir moved outward and completely re-solvated. The final interaction energy at 250 ns was 0.65 kcal/mol (non-polar: 0.53 kcal/mol, polar: 0.12 kcal/mol), indicating complete ritonavir dissociation from HIVp.

Ritonavir lost 55-fold of binding affinity towards HIVp with mutations of L10I, G48V, I54V, L63P and V82A [47]. Notably, both I54V and V82A mutants are located on the flap/loop pathway with smaller side-chains, which would inevitably reduce the VDW interaction between HIVp and ritonavir, leading to a faster dissociation process.

Different from ritonavir interacting with the loop region during dissociation, xk263 spent most of the time in the bound state, owing to the strong H-bond network with flaps and catalytic triads (Figure 5a and Figure S3). Such an intermolecular H-bond network was mainly restricted to residues Asp 25, Ile 50, Asp 124 and Ile 149 because the H-bond length was 1.85 Å for xk263–Asp 25 and 1.78 Å for xk263–Ile 50. Interaction energy between xk263 and HIVp at 0 ns was −92.03 kcal/mol (non-polar: −123.27 kcal/mol, polar: 31.24 kcal/mol), which is 15.92 kcal/mol stronger than that of ritonavir in the bound state. During unbinding, xk263 tilted at 86 ns, which led to decreased interaction between xk263 and HIVp as flap A lifted and the diol group was released from the catalytic triads, thus resulting in an interaction energy barrier of −83.84 kcal/mol (non-polar: −107.58 kcal/mol, polar: 24.15 kcal/mol) (Figure 5b). Even though xk263 moved back to the binding pocket at 104 ns (Figure 5c), the flap handedness was disrupted, with only one flap contacting xk263 and the ligand RMSD increased (Figure 3(A,ii)). Xk263 was trapped inside the pocket until 440 ns, when xk263 moved toward chain B and formed an H-bond with Pro 180 and Val 181 with interaction energy of −62.94 kcal/mol (non-polar: −82.87 kcal/mol, polar: 19.93 kcal/mol) (Figure 5d) and H-bond length of 1.77 Å and 1.70 Å, respectively. Finally, flap B opened (Figure 5e), which allowed xk263 to shift into the flap/loop region, followed by both flaps opening and xk263 continuing to unbind (Figure 5f).

#### 3.1.2. Pathway B: Dissociation with Surface Diffusion through the Flap Region

Pathway B is defined as the ligand unbinding along the flap without contacting the loop region (Figure 2b). Four of 20 ritonavir and zero xk263 dissociation trajectories used this pathway. Xk263 has four aromatic groups, and the hydrophobicity always drives the ligand to contact with the loop region. Similar to pathway A, during ritonavir dissociation under pathway B, flaps fluctuated spontaneously, which can be irrelevant to the position of ritonavir and not directly induced by ritonavir (Figure 3(B,i)). However, ritonavir moved with one flap as the flaps moved open/closed at ~280 ns, thus resulting in increased ligand RMSD fluctuation. Because it moved with a flap, ritonavir also underwent more noticeable rotation (Figure 6e) as compared with the conformations found in pathway A. Nevertheless, ligand rotation did not affect protein motions, and the RMSF of HIVp in both pathways A and B had major fluctuations from the flap region (Figure 3(A,iii) and (B,ii)).

In the beginning of the simulation (see the same conformation as Figure 4a), a stable H-bond between ritonavir and Asp 29 (bond length 1.80 Å) remained until 75 ns (Figure S4). The energy fluctuated when ritonavir stayed close to the crystal structure bound complex (Figure 3(B,i) before 55 ns). At 179 ns (Figure 6b), ritonavir moved in between flaps A and B, where a H-bond with Asp 29 and subsequently with Ile 50 was broken, thus resulting in a slightly increased interaction energy of −81.72 kcal/mol (non-polar: −117.74 kcal/mol, polar: 36.03 kcal/mol). Ritonavir continued wiggling, and at 213 ns, the drug formed a transient H-bond with Asp 128 (length 1.86 Å) to create a local energy minimum by reducing the polar interaction (total energy: −102.52 kcal/mol; non-polar: −121.91 kcal/mol, polar: 19.93 kcal/mol). At 279 ns, the flaps were fully open, and ritonavir maintained contact with flap A only, which significantly weakened the non-polar interaction energy (total: 57.33 kcal/mol; non-polar: −64.58 kcal/mol, polar: 18.51 kcal/mol) (Figure 6d). Then ritonavir migrated to the outer side of flap region, partially solvated in water raising the interaction energy to −5.12 kcal/mol (Figure 6e) followed by further ligand rearrangement and complete unbinding.

Previous studies reported that some drug resistance mutations are distal from the ligand binding site [3,47,48]. It is therefore not straightforward to explain how the mutations affect inhibitor binding. We noticed residues 43 and 46 with major mutations of K43T and M46IL are located on pathway B, and ritonavir formed H-bonds with Lys 43 and Met 46 before the drug completely dissociated from HIVp (Figure S4, chain B residues 142 and 145). Although future investigations are needed to quantify the contribution in binding energy if Lys 43 and Met 46 mutate to other residues, our simulations suggested that the two amino acids are important in holding ritonavir under pathway B.

#### 3.1.3. Pathway C: Dissociation with Surface Diffusion through Interface Region

Pathway C is defined as a ligand diffusing on the interface region and then unbinding from HIVp (Figure 2c). One unique HIVp motion in this pathway is that the flaps open widely at first and then ligand dissociation occurs. Dissociations under pathway C were observed in 3 of 20 ritonavir trajectories and 3 of 15 xk263 trajectories. It took longer time (543 ns) for ritonavir to unbind from HIVp under pathway C as compared with pathway A (249 ns) or pathway B (305 ns), whereas xk263 spent a similar time dissociating under pathways A and C. Wide-open flap handedness was observed before ligand unbinding in both ritonavir and xk263 dissociation under this pathway (Figure 7c and Figure 8d). For example, ritonavir shifted to the catalytic triad and diffused along the interface region when flap tip distance was > 30 Å after 479 ns (Figure 3(C,i)). Similarly, xk263 moved to the interface region with noticeable flap openness after 453 ns (Figure 3(C,ii)). Even after leaving the binding pocket, ligands may remain in the interface region for a significantly long time, which is consistent with existing studies that this area is a highly favorable non-specific binding site for ligands [49]. As a result, only one of three ritonavir trajectories and one of three xk263 trajectories achieved complete dissociation from HIVp under pathway C. Notably, because no stable H-bonds were observed when a ligand stays in the interface region (Figures S5 and S6), the non-specific attraction was mostly non-polar interactions.

Initially, the crystal structure bound complex (Figure 4a) had a stable H-bond between ritonavir and Asp 29, but the H-bond broke at ~40 ns. Two new H-bonds between Ile 50 and Arg 107 formed, which strengthened the $∆E_{\mathrm{polar}}$ and lasted 50 ns (Figure 7a and Figure S4). However, $∆E_{\mathrm{MM}/\mathrm{PBSA}}$ kept increasing because vdW contacts were reduced when ritonavir gradually moved away from the crystal structured bound form until 53 ns, when ritonavir interacted with five non-polar residues (Ile 50, Ile 84, Val 131, Pro 180 and Val 181 in Figure 7a) to bring the system to a local energy minimum (total energy: −100.25 kcal/mol; non-polar: −119.97 kcal/mol, polar: 19.38 kcal/mol). Ritonavir stayed in the region until 245 ns (Figure 7b), when the drug curled within the binding pocket and reduced the contacts with HIVp, thus yielding an energy barrier of −61.14 kcal/mol (non-polar: −97.64 kcal/mol, polar: 36.50 kcal/mol). Figure 7c shows that at 434 ns, both flaps opened, and ritonavir arranged to an extended form, thus resulting in a local energy minimum of −100.63 kcal/mol (non-polar: −125.02 kcal/mol; polar: 24.40 kcal/mol). The flaps continued opening widely and ritonavir formed contacts with only the interface region (Figure 7d), which quickly increased interaction energy to −52.10 kcal/mol (non-polar: −61.47 kcal/mol, polar: 9.36 kcal/mol). Ritonavir stayed in the area for ~60 ns before it eventually detached from the interface region as interaction energy reached ~0 kcal/mol. In the other two ritonavir dissociations under pathway C, ritonavir may attach to the interface region for longer than ~125 ns (equivalent to five re-seeding attempts) and yet no complete unbinding was observed.

Similar to xk263 unbinding from pathway A (see the same conformation as Figure 5a), the ligand formed a stable H-bond network at the beginning of the aMD run (Figure S6). Xk263 jiggled inside the binding pocket until it exposed a diol group toward solvent at 222 ns with interaction energy of −79.21 kcal/mol (non-polar: −101.58 kcal/mol, polar: 22.37 kcal/mol) (Figure 8a), followed by partially sliding out of the binding pocket and partially shifting to the flap/loop region with greatly decreased non-polar interaction energy, −86.14 kcal/mol at 264 ns (Figure 8b). Flap A opened up at 452 ns, whereas xk263 maintained interactions with flap B and moved back to interact with the interfacial region, which enhanced the non-polar interaction to −110.21 kcal/mol (Figure 8c). Xk263 moved with flap B to the flap/loop region at 486 ns (Figure 8d). However, the complex failed to keep strong attractions (total energy: −43.66 kcal/mol; non-polar: −54.01 kcal/mol, polar: 10.35 kcal/mol), and xk263 quickly shifted to the interface region with the diol group contacting HIVp and the ketone group exposed to solvent (Figure 8e). In this sampled dissociation trajectory, xk263 continued to diffuse on the interface region for longer than 100 ns (equivalent to four re-seeding attempts, which are not included here for the trajectory length). If xk263 successfully unbound from HIVp, xk263 spent only ~20 ns contacting the interface region.

#### 3.1.4. Other Pathways

Besides the three major dissociation pathways, ritonavir could solvate and unbind when flaps were wide open (observed in one trajectory) or attach to the flap tip when the flap opened to dissociate (one trajectory). Xk263 could unbind through the gap between flaps (one trajectory), move with the flap tip (one trajectory) or directly slip out of the binding pocket with the closed-flap HIVp conformation (two trajectories).

Existing studies showed that xk263 can bind to HIVp with semi-open flaps [11,27]; therefore, here we discuss a case in which xk263 dissociated with slightly-open flaps and closed-flap handedness. Only 2 of 15 xk263 dissociation trajectories showed closed-flap handedness during unbinding. Among them, one trajectory completed unbinding and the other remained on the protein surface when we terminated the run. Before xk263 left the pocket, HIVp had slightly-open flap handedness during ligand rearrangement inside the binding pocket, but the flaps never fully opened. None of the 20 ritonavir dissociation trajectories had closed flap handedness. Because of the special flap behavior, we classified this pathway under “other pathways” and discussed it in the following text. Flaps remained closed at ~5 Å, but a significant xk263 RMSD increase was recorded starting at 300 ns (Figure 3(D,i)). HIVp also experienced less fluctuation because the backbone RMSF was <3.8 Å as compared with 5.4 Å for pathway A (Figure 3(A,iii)) and 5.7 Å for pathway C (Figure 3(C,iii)). An important precondition for this pathway was that xk263 flipped upside down within the binding pocket when flaps opened at 50 ns and water molecules entered the binding pocket to weaken the interactions between xk263 and HIVp (Figure 9b). H-bond analysis revealed that only 13 residues formed an H-bond with xk263, but there were 22 residues in pathway A and 19 residues in pathway C, respectively (Figures S3, S6 and S7).

Xk263 remained inside the bonding pocket until the flap region slightly opened, which allowed xk263 to flip upside down. During xk263 flipping at 63 ns, the non-polar interaction was slightly weakened, from −130.04 kcal/mol to −120.68 kcal/mol, thus resulting in a small energy barrier of −97.09 kcal/mol. The H-bond between xk263 and HIVp barely changed before and after ligand flipping (Figure S7) because a carbonyl oxygen in xk263 formed H-bonds with Ile 50/Ile 149 and two hydroxyl groups formed H-bonds with Asp 25/Asp 124 before flipping. After xk263 flipping, carbonyl oxygen formed H-bonds with Asp 25/Asp 124 and two hydroxyl groups formed H-bonds with Ile 50/Ile 149. The flaps moved from slightly-open to a closed conformation and xk263 remained in the flipped position with bridge waters observed between the flaps and the catalytic triads at 97 ns (Figure 9b). The two bridge water molecules joined the H-bonds between xk263-HIVp at about 100 ns as the H-bonds (xk263 and Asp 25, Asp 124, Ile 149) broke and reformed (Figure S7). From 50 ns to 280 ns, the RMSD of flipped xk263 remained steady at ~10 Å, which suggests that the bridge waters stabilized xk263 (Figure 3(D,i)). At 294 ns, xk263 exposed the diol group to solvent for unbinding (Figure 9c), then xk263 moved into flap A and the loop region. Because the flaps were closed, xk263 remained in contact with flap B, but the attractions were weak (total energy: −69.77 kcal/mol; non-polar: −99.75 kcal/mol, polar: 29.98 kcal/mol) at 313 ns. Xk263 diffused on the HIVp surface after it passed through the flap/loop region. Because previous studies suggested that the surface diffusion may last longer than 1 µs, we terminated the simulation because xk263 completely left the binding pocket.

Traditional computer-aided drug design (CADD) mostly focuses on ligand-protein bound-state and assumes that there is only one dominant ligand binding/unbinding pathway for inhibitor binding. As a result, it is puzzling how several distal mutations located in various locations of HIVp associate with resistance to inhibition. Our study revealed that HIVp inhibitors have multiple unbinding pathways. Mutations far from the inhibitor binding site may hamper stable intermediate states during drug unbinding which prolong dissociation time and is helpful to inhibit protein function. Pathways sampled here provided insights into why distal mutations are not clustered in certain locations and suggested that different ligand unbinding processes can be affected by different mutated residues.

### 3.2. Association–Dissociation Trajectories Comparison

To examine the similarity of ligand binding/unbinding processes, we first investigated the mutual protein conformations during ligand binding and unbinding. Comparing protein backbone RMSD between different frames from various trajectories is a simple and precise strategy to identify similar protein conformations. We selected the dissociation trajectories of pathway A for ritonavir (Figure 2a and Figure 4) and xk263 (Figure 5) and re-saved a frame every 1 ns, yielding a total of 248 and 479 reference frames, respectively. Each frame served as a reference structure to calculate protein backbone RMSD for the association trajectories. In comparison with a reference frame, if the computed RMSD between two structures was < 2.5 Å, we considered it an overlapping HIVp conformation with the reference structure.

Both ritonavir and xk263 preferred the flap/loop pathway, dissociation pathway A, when unbinding, as shown above (Table 1). Previous studies also showed that HIVp ligands with different molecular properties prefer different association pathways and/or distinct association rate constants [24,29,50]. However, whether their unbinding processes are simply the reverse process of ligand binding is unclear. Notably, classical binding mechanisms such as conformation selection and induced-fit models mainly describe the conformational fluctuations during ligand binding. For example, ritonavir binds to HIVp with a conformation selection model, whereas xk263 binds with an induced-fit model [27]. Therefore, we wondered whether xk263 could induce the same HIVp motion in both binding and unbinding processes.

**Figure 10:**
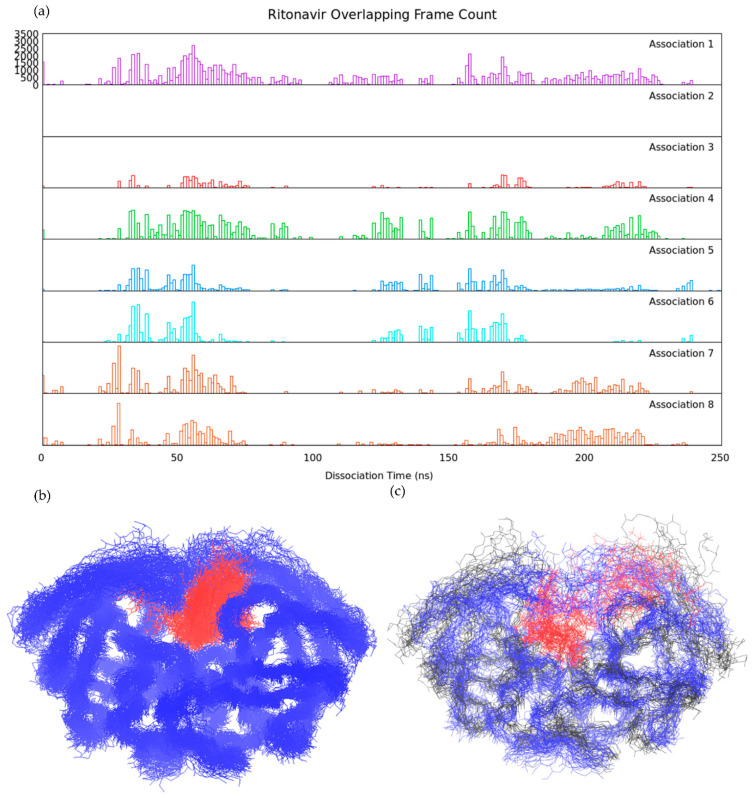
Ritonavir association-dissociation comparison under pathway A. (**a**) Distribution of overlapped frame (< 2.5 Å RMSD) count from eight ritonavir association trajectories compared to ritonavir dissociation. The reference frame was taken every 1 ns from the dissociation trajectory. (**b**) Ritonavir-HIVp conformations with overlapped frame in seven association trajectories. Ritonavir-HIVp was horizontally flipped to better visualize ritonavir’s position. (**c**) Ritonavir-HIVp conformations with no overlapped frame in any association trajectories. HIVp is shown in blue line with backbone. Wide-open HIVp conformations are shown in black line. Ritonavir is shown in red line.

In the ritonavir dissociation–association comparison, overlapping conformations were found in seven of eight association trajectories (Figure 10a). Association #2 had no frame similar to any reference frame in the compared dissociation trajectory because HIVp experienced uncommonly large elbow region displacement during ritonavir association (Figure S8). The population of overlapping frames revealed that HIVp conformations at ~50, ~170 and ~220 ns from the dissociation trajectory were popular among association trajectories (Figure S9). HIVp flap conformations were semi-open or slightly-open with a flap distance of 6.91 Å, 7.23 Å and 5.96 Å for snapshots at 50 ns, 170 ns and 220 ns, respectively. Ritonavir spent most of the time inside the binding pocket with flaps closed or semi/slightly-open during dissociation. The two-step binding mechanism suggests that the ligand–HIVp spends a long time in conformational change to form a tighter complex [25,46]. Existing studies suggested that the semi-open flap conformation was purposed to be the most stable intermediate state during the flap open/closed position [51,52], which also explains why semi-open/slightly-open flaps were commonly observed in both ligand binding/unbinding in our trajectories. HIVp conformations in ~10 ns, ~100 ns and ~240 ns are not common in association trajectories because the frame count is low or none (Figure S10). Elbow loop distortion at 10 ns was the main reason why we found no overlapping conformation at this step, even though the flaps were in a slightly-open conformation (i.e., flap tip distance ~6.09 Å). Once the elbow loop recovered from distortion, more overlapping conformations were found starting at ~25 ns. HIVp flap conformations were wide open, with flap distance of 17.56 Å and 27.57 Å for snapshots at 100 ns and 240 ns, respectively. The wide-open conformations were less popular, which agrees with a previous study reporting ~6 kcal/mol higher conformational free energy of substrate-HIVp with open-flap than semi-open flap conformation [46]. Hence, HIVp tends to remain in semi/slightly-open flap conformation.

With a total of 248 reference frames in ritonavir dissociation trajectory, 98 frames in dissociation pathway A were found to be overlapping conformations in all seven association trajectories (Figure 10b), whereas 31 reference frames had no overlapping frame in any association trajectories (Figure 10c). In these 98 frames, ritonavir mostly located between the flap/loop region, and HIVp flaps were slightly-open. According to the conformational selection model, these 98 frames are selected popular conformations when ritonavir is entering/leaving the binding pocket. In contrast, when HIVp flaps were wide open, even when ritonavir also located in the flap/loop region, these nine conformations (black lines shown in Figure 10c) did not overlap with any frames in association. In the other 22 no-overlapping conformations, ritonavir located inside the binding pocket, attaching to a loop region while HIVp flaps were closed, accompanied by large elbow displacement or open, accompanied by a distorted flap tip. We can conclude that HIVp constantly fluctuates during ritonavir dissociation, yet there are certain HIVp conformations that occur in almost all ritonavir association trajectories. Commonly preferred protein conformations during ritonavir association or dissociation exist, which suggests that the drug utilizes a conformational selection model for both binding and unbinding to HIVp.

**Figure 11:**
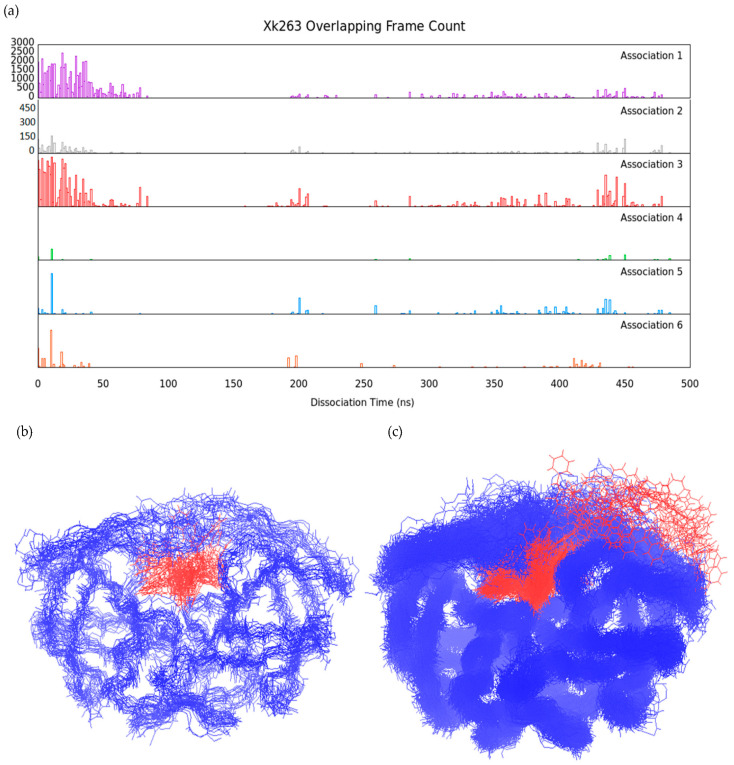
Xk263 association–dissociation comparison under pathway A. The y-axis range is 0–3000 for association 1 and 0–450 for the rest of the association trajectories. (**a**) Distribution of overlapped frame (<2.5 Å RMSD) count from six xk263 association trajectories compared to xk263 dissociation. The reference frame was taken every 1 ns from the dissociation trajectory. (**b**) Xk263-HIVp conformations with overlapped frame in all association trajectories. (**c**) Xk263-HIVp conformations with no overlapped frame in any association trajectories. HIVp is shown in blue line with backbone only. Xk263 is shown in red line.

In contrast to ritonavir, with thousands of overlapping frames during association/dissociation, xk263 association-dissociation shows significantly fewer overlapping conformations (Figure 11a). The distribution of frame count revealed that HIVp conformations at ~10 ns, ~200 ns and ~440 ns were popular in association trajectories with flap distance 9.03 Å, 11.33 Å and 7.46 Å, respectively (Figure S11). HIVp in xk263 dissociation favors slightly/semi-open flap conformations than wide-open conformations because wide-open conformations are at high energy states. Of note, even in the bound state (snapshot at 10 ns), the xk263-HIVp complex had fewer overlapping frames than ritonavir-HIVp, which suggests that xk263 introduced various HIVp conformational changes. Unique HIVp conformations were induced by xk263 unbinding, such as frames between 90 to 190 ns and snapshots at 100 ns, 140 ns and 160 ns shown in Figure S12, where the flaps stacked above xk263, and the elbow loop was largely distorted (Figure S13). Here, flap stacking refers to an asymmetric motion where one flap remains contacting with xk263 while the other flap rotates away from xk263. Flap stacking was only observed in the xk263 dissociation trajectory where the carbonyl group of xk263 tightly binds to one flap tip and the catalytic trials move together with xk263 (Figure S13). The concerted motions result in the unique conformations in xk263 dissociation, which further demonstrates that xk263-HIVp undergoes an induced-fit mechanism during unbinding. With a total of 479 reference frames, 18 frames in xk263 dissociation were overlapping conformations in all six association trajectories (Figure 11b), whereas 214 frames had no overlapping frame in association trajectories (Figure 11c). In these 18 frames, xk263 mostly located inside the binding pocket and HIVp flaps were closed. Unlike ritonavir, xk263 quickly moved through the flap/loop region to dissociate; we did not observe popular HIVp conformations with xk263 between the flap/loop region. Figure 5g shows a small energy fluctuation during xk263 unbinding, which suggests a constantly induced small conformational rearrangement during ligand dissociation. The local environment during xk263 binding and unbinding is not the same. As a result, the conformations induced during xk263 association–dissociation are rare.

The overlapping HIVp conformation frame count difference between ritonavir and xk263 dissociation supported a conformational-selection mechanism for ritonavir-HIVp and induced-fit mechanism for xk263-HIVp. With ritonavir binding/unbinding from HIVp under pathway A, protein conformations with ritonavir between the flap/loop were observed in both dissociation and association. In contrast, HIVp conformations rarely overlapped during xk263 association/dissociation, which suggests that the conformations were induced during xk263 association/dissociation. The environment during xk263 binding/unbinding is not identical, which results in different induced conformations.

### 3.3. Mutual Conformations in Association/Dissociation

Here, we discuss the pathways in detail to understand the similarities and differences between ritonavir and xk263 during unbinding.

#### 3.3.1. Closed Flap Conformation

HIVp with a closed-flap configuration was the most-observed mutual conformation in the ritonavir dissociation–association comparison, which was expected because all dissociation trajectories started with a closed flap configuration and all association trajectories ended with a closed or semi-open configuration. In contrast, only one reference conformation with a closed flap in xk263 dissociation was found with similar HIVp conformations in xk263 association 1.

**Figure 12:**
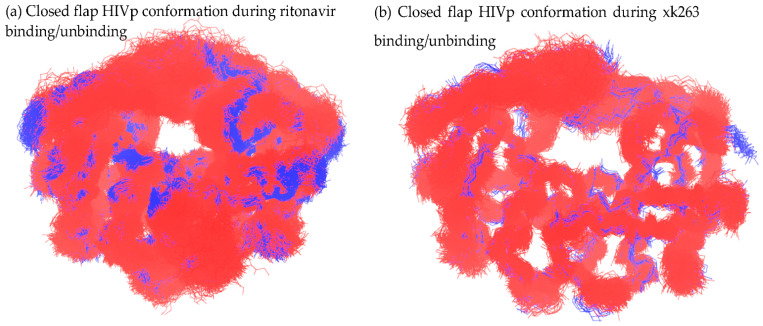
Overlapped frames with closed flaps and projections of HIVp configurations on PC space. (**a**) HIVp conformations in ritonavir dissociation trajectory under pathway B (red) using Figure 6a as a reference frame and corresponding overlapped frames from ritonavir association 1 (blue). (**b**) HIVp conformations in xk263 dissociation trajectory under pathway C (red) using Figure 8a as a reference frame and corresponding overlapped frames from xk263 association 1 (blue).

Using the initial frame of ritonavir dissociation trajectories as a reference frame (Figure 4a), we observed the overlapping conformation in seven association trajectories with abundant frame counts. Figure 4a was the initial frame of aMD simulation, and the protein conformation is nevertheless highly similar to its crystal structure conformation. Therefore, we selected another closed-flap HIVp conformation in pathway B (Figure 6a, the frame taken in 32 ns) as a reference structure and found 1283 overlapping frames from our reference dissociation pathway B and 505 overlapping frames from association 1 (Figure 12a). Hundreds of similar conformations with closed flaps on ritonavir association/dissociation suggested that the pre-existing conformations are selected during ritonavir binding/unbinding processes, which implies use of a conformational-selection binding mechanism.

Using eight xk263-HIVp conformations with a closed flap from previous figures (Figure 5b,c, Figure 8a,b and Figure 9a–d) to examine conformation overlap with xk263 association 1, we found that only 27 frames overlapped with conformations (Figure 8a). No conformations from association 1 were similar to the other seven closed-flap conformations that appeared during xk263 dissociation. Using the HIVp conformation in pathway C (Figure 8a) as a reference structure, we found 2710 overlapping frames from a dissociation trajectory with 4932 frames, but only 27 overlapping frames from the association 1 trajectory with 3000 frames (Figure 12b). Although Figure 8a is a highly populated conformation in pathway C, the low similarity of closed-flap confirmations between xk263 dissociation and association raised our interest. The low similarity is due to the induced-fit model with different micro-environments during xk263 association and dissociation precluding the system from inducing similar HIVp conformations.

In the ritonavir-HIVp bound complex, a bridge water molecule connects ritonavir and the flap region with H-bonds [33,35]. The cyclic-urea inhibitors were designed by using the oxygen atom in the carbonyl group of cyclic urea to replace the bridge water [34]. The diol group in xk263 forms H-bonds with catalytic triads, especially with catalytic residues Asp 25 and Asp 124. Hence, the H-bond network consisting of the flap region-xk263-catalytic triads tightly clamped xk263 in a non-polar environment. The H-bond network and non-polar binding pocket also restricted flap motions, as seen in Figure 3, in which flap distance is generally less in xk263 unbinding than ritonavir unbinding. As compared with xk263 association, in which the ligand was binding to a pocket full of water molecules, the ligand was unbinding from a hydrophobic environment, which induced significantly different HIVp conformations during association–dissociation. Our analysis suggests that an induced-fit mechanism is used for xk263-HIVp dissociation as well.

#### 3.3.2. Open Flap Configuration

HIVp with an open-flap configuration was observed in both ritonavir and xk263 dissociation trajectories. As a ligand moves to one flap/loop region, the ligand formed an asymmetric contact with the flaps, thus resulting in the flaps opening.

**Figure 13:**
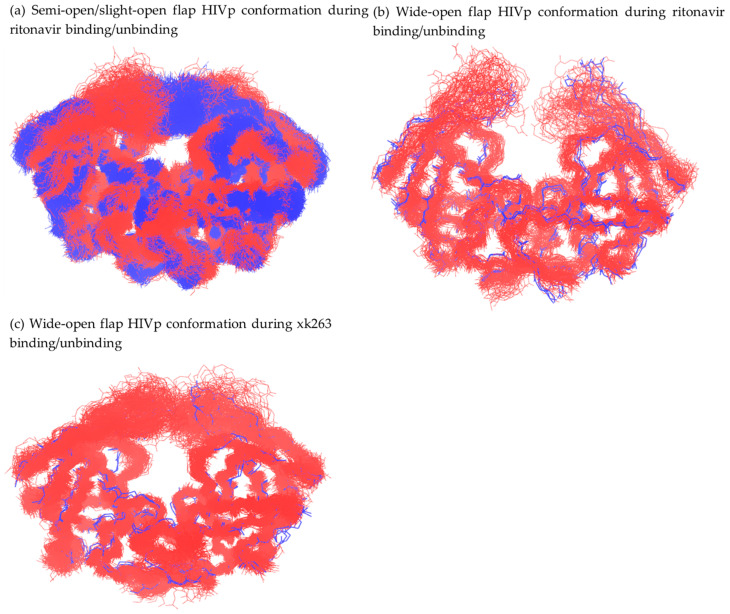
Overlapped frames with open flaps and projections of HIVp configurations on PC space. (**a**) HIVp conformations in ritonavir dissociation trajectory under pathway A (red) using Figure 4b as a reference frame and their corresponding overlapped frames from ritonavir association 1 (blue). (**b**) HIVp conformations in ritonavir dissociation trajectory under pathway A (red) using Figure 4c as a reference frame and their corresponding overlapped frames from ritonavir association 1 (blue). (**c**) HIVp conformations in xk263 dissociation trajectory under pathway A (red) using Figure 5f as a reference frame and their corresponding overlapped frames from xk263 association 1 (blue).

Here we used a slightly-open (Figure 4b, 34 ns) and wide-open conformation (Figure 4c, 172 ns) from the most popular ritonavir dissociation pathway A to examine association–dissociation overlapping. We used the flap tip distance (α-C distance between Ile 50-Ile 149) to define slightly (7.70 Å in Figure 4b) or wide open (14.92 Å in Figure 4c) conformations. Different flap openness yielded significantly different results. In the association 1 trajectory, 1803 and 3 overlapping frames had a slightly-open (Figure 13a) and wide-open (Figure 13b) conformations, respectively. Notably, the trajectory recorded a total of 3000 frames; therefore, this overlapping conformation populated 60.2% of the entire association process. Comparing the entire dissociation pathway A with frame 4b of the same trajectory, this conformation is also highly popular: 12.94% of the entire ritonavir dissociation process. Notably, this slightly-open conformation is also one of the most popular conformations when HIVp is in the ligand-free state, which suggests that ritonavir also selects this conformation during dissociation [27].

Wide-open conformations also pre-exist in the ligand-free state, but they are significantly less populated because of unfavorable conformational free energy. Previous studies showed that conformational free energy for HIVp was ~6 kcal/mol higher for wide-open than semi-open confirmations [46]. As compared with the wide-open conformation (Figure 4c) with the entire dissociation pathway A, only 54 frames (2.17%) overlapped with this wide-open conformation. Because of the free energy cost and highly flexible open flaps, only a few (i.e., three in this case) overlapping frames from association 1 to Figure 4c were anticipated.

As for xk263, we also selected a slightly-open (Figure 5d, 440 ns) and wide-open (Figure 5f, 467 ns) flap to examine conformation overlapping during xk263 association-dissociation. Regardless of flap openness, in the association 1 trajectory, we found zero and two overlapping frames with a slightly-open and wide-open conformation, respectively (Figure 13c). Although a low overlap possibility with a wide-open conformation during association-dissociation is anticipated, we did not expect that no frames from association 1 would show the similar slightly-open HIVp conformation observed in dissociation pathway A. Therefore, we checked the population of the two open conformations in pathway A; slightly-open conformations (Figure 5d) occupied 41.4% (1989 of 4800 frames) of the entire trajectory and wide-open conformations 12.5% (600 of 4800 frames). Even though the slightly-open conformation was highly popular in dissociation pathway A, the induced flap conformations were unique in xk263 dissociation. As a result, the wide-open conformation with symmetric flap opening is the only common open HIVp conformation during xk263 association–dissociation. Xk263 is highly hydrophobic. Because the pocket of HIVp contains mainly non-polar residues, xk263 prefers contacting with HIVp to induce new protein motions. For example, when xk263 moved to flap B/loop B for dissociation, xk263 could also remain in contact with flap A, as seen in Figure 5d,e for pathway A and Figure 8d for pathway C. As xk263 continued dissociating, xk263–flap A interactions promoted asymmetric flap A movement, thus resulting in a newly induced HIVp flap conformation. Hence, new HIVp conformations were constantly induced as xk263 was unbinding from the pocket. Unlike ritonavir, which can partially re-solvate after flaps open widely (Figure 4c and Figure 7d), xk263 always favors contacting with flap/loop regions instead of easily re-solvating into the solvent. We also believe that the hydrophobicity of xk263 prevents the ligand from undergoing pathway B because diffusion on the surface through the flaps exposes most of the ligand to solvent. As suggested in previous papers, ligand properties contribute to the binding mechanisms, and here we showed that the properties affect unbinding mechanisms as well [10,17,27].

## 4. Conclusions

The study used unbiased aMD and a re-seeding approach to sample unbinding pathways of ritonavir-HIVp and xk263-HIVp, which brings a more comprehensive picture for understanding molecular recognition and unbinding mechanisms of ligand and protein. We observed three common dissociation pathways: between the flap/loop region, diffusion on the flap region, and diffusion on the interface region. Dissociation between flap/loop regions (pathway A) was the major dissociation pathway for both ritonavir and xk263 because of strong non-polar interactions between the ligand and HIVp to bring increased contacts between the ligand and loop region during dissociation. However, the loop region could form transient but strong H-bonds with ritonavir to prevent ritonavir from dissociation. The H-bond between the loop region and xk263 has a short lifetime but helps to stabilize xk263. Diffusion on the flap, pathway B, with the ligand moving to the solvent following the flap opening, was observed only in ritonavir dissociation. The non-polar property of xk263 keeps xk263 contact with HIVp even after the flaps already opened. Diffusion on the interface region was observed in both ritonavir and xk263 dissociation.

We observed overlapping conformations between dissociation and association trajectories for both ritonavir and xk263 unbinding/binding. However, overlapping HIVp conformations are rarely seen between xk263-HIVp association/dissociation. Because the environments during binding/unbinding differ, significantly different conformations are induced during unbinding. Previous studies suggested a conformational-selection binding mechanism for ritonavir. During unbinding processes, ritonavir revisited these conformations, which suggests the use of a conformational-selection mechanism in unbinding as well. Among overlapping conformations, we observed more closed-flap conformations than slightly-open or wide-open conformations for both ritonavir and xk263. In general, ritonavir requires open flaps to achieve unbinding, whereas xk263 can unbind with a closed-flap conformation. Our study suggests that traditional structure-based drug design that focused on the bound state may be extended to stabilizing the transient conformations during ligand binding/unbinding to prolong drug dissociation and increase the dissociation rate constant.

## Figures and Tables

**Table 1:** Ritonavir and xk263 dissociation trajectories sorted by pathways.

	Ritonavir	xk263
Total dissociations	20	15
Pathway A: Unbind between flap/loop	11	8
Pathway B: Diffusion on flap	4	Not observed
Pathway C: Diffusion on interface	3	3
Others	2	4

## Data Availability

The data presented in this study are available on request from the corresponding author. The data are not publicly available due to large file size.

## Supplementary Materials

The following are available online at https://www.mdpi.com/article/10.3390/life12010116/s1.
