# Supplementary material for: Ritonavir and xk263 Binding-Unbinding with HIV-1 Protease: Pathways, Energy and Comparison
Source: Life (Basel). 2022 Jan 13;12(1):116. doi: 10.3390/life12010116 (PMC8779838; doi:10.3390/life12010116)
Supplement: Supplementary file 1 [file life-12-00116-s001.zip › life-1525487-supplementary.pdf]

life

MDPI

Supplementary Materials: Ritonavir and xk263 Binding-Unbinding with HIV-1 Protease: Pathways, Energy and Comparison

Table S1. Boost potentials for ritonavir and xk263 dissociation in reseeding approach under accelerated molecular dynamic simulations. where ED and EP are average dihedral energy threshold and average total potential energy threshold, respectively. αP and αD are boost factors for the dihedral potential and total potential, respectively. Boost factors were calculated based on 100 ns bound state ritonavir-HIVp and xk263-HIVp cMD simulations.

Boost potential (kcal/mol)	Ritonavir	Xk263
EhreshD (ED)	3184	3341
alphaD (αD)	159	159
EthreshP (Ep)	−92163	−96460
alphaP (αP)	5204	5439

Table S2. Reseeding attempts to achieve successful ligand dissociation for ritonavir-HIVp and xk263-HIVp systems. Five 25 ns simulations were carried in each attempt. Xk263 required more attempts to dissociate when compared to ritonavir.

Ritonavir-HIVp	Attempts	Pathway	Xk263-HIVp	Attempts	Pathway
Seed 01	2	Other	Seed 01	3	C
Seed 02	3	A	Seed 02	7	A
Seed 03	3	A	Seed 03	12	A
Seed 04	2	A	Seed 04	7	A
Seed 05	5	B	Seed 05	7	A
Seed 06	4	A	Seed 06	5	Other
Seed 07	5	A	Seed 07	8	C
Seed 08	5	B	Seed 08	10	A
Seed 09	1	Other	Seed 09	13	Other
Seed 10	5	A	Seed 10	14	C
Seed 11	5	C	Seed 11	12	A
Seed 12	3	C	Seed 12	15	Other
Seed 13	1	A	Seed 13	12	A
Seed 14	1	B	Seed 14	9	A
Seed 15	6	A	Seed 15	10	Other
Seed 16	4	A			
Seed 17	3	B			
Seed 18	6	C			
Seed 19	4	A			
Seed 20	1	A			

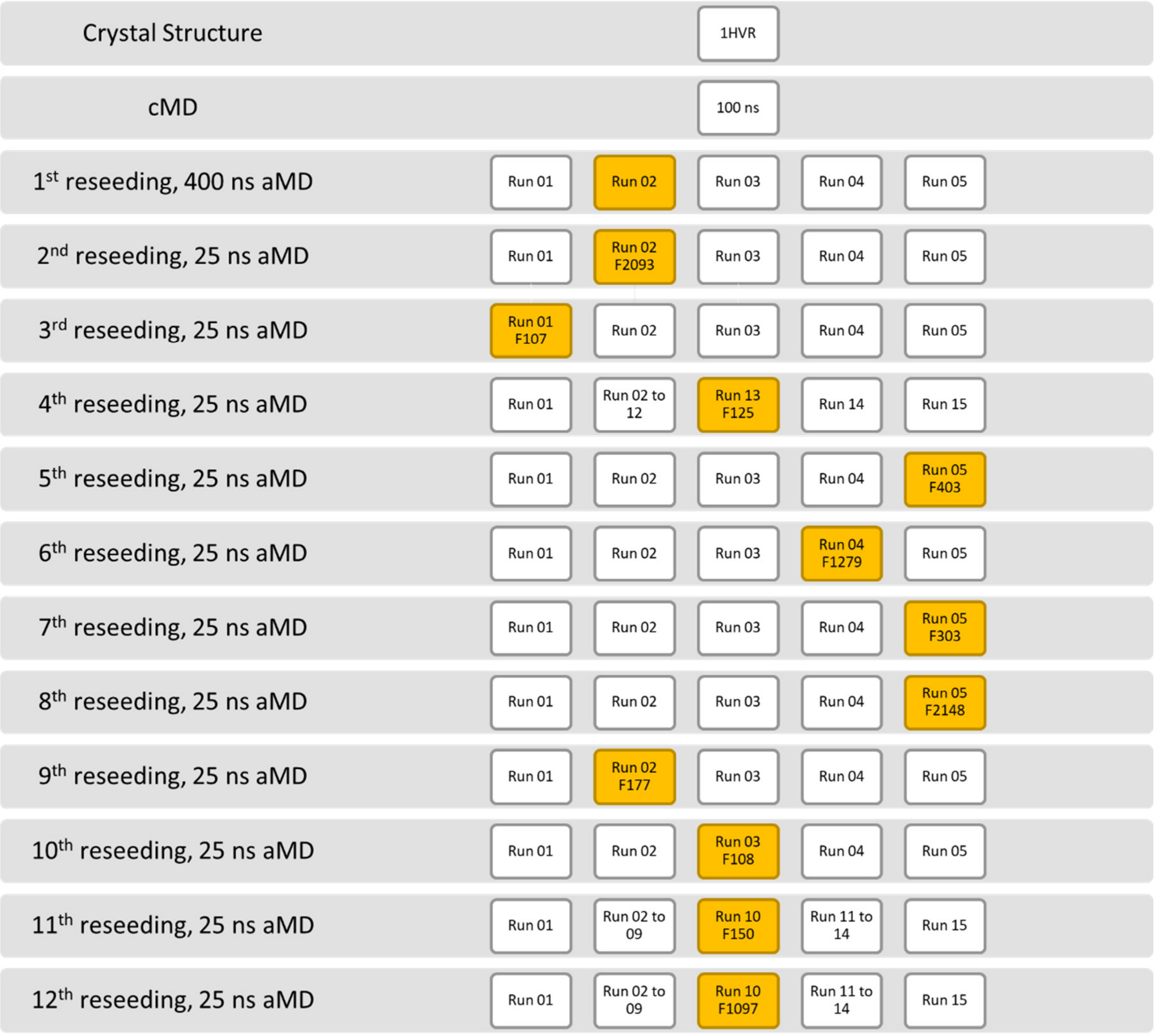

Figure S1. Reseeding approach details for xk263 dissociation seed 3. Highlighted cell is the selected seed and the selected frame indicated. Using the last frame from cMD as initial frame, we performed first reseeding with five 400 ns long aMD simulations and selected run 02. Using the last frame from first reseeding run 02, we performed second reseeding with five 25 ns long aMD simulations and selected frame 2093 in run 02. Time interval between frames is 10 ps. We obtained a total of 47990 frames aMD simulation, which equals 479.9 ns.

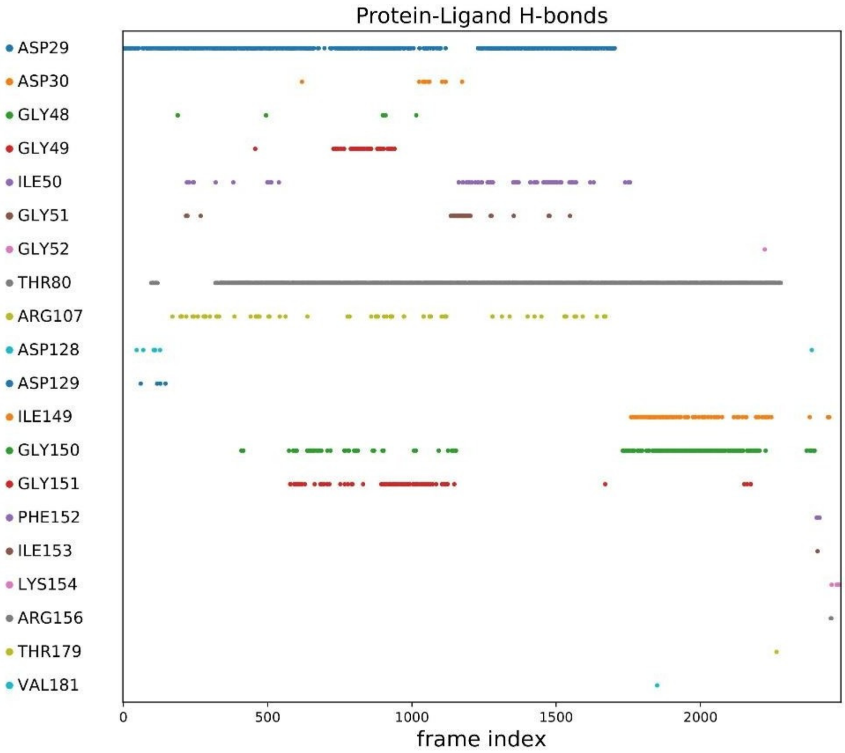

Figure S2. Hydrogen bond plot for ritonavir-HIVp under Pathway A dissociation. Time interval between each frame is 0.1 ns.

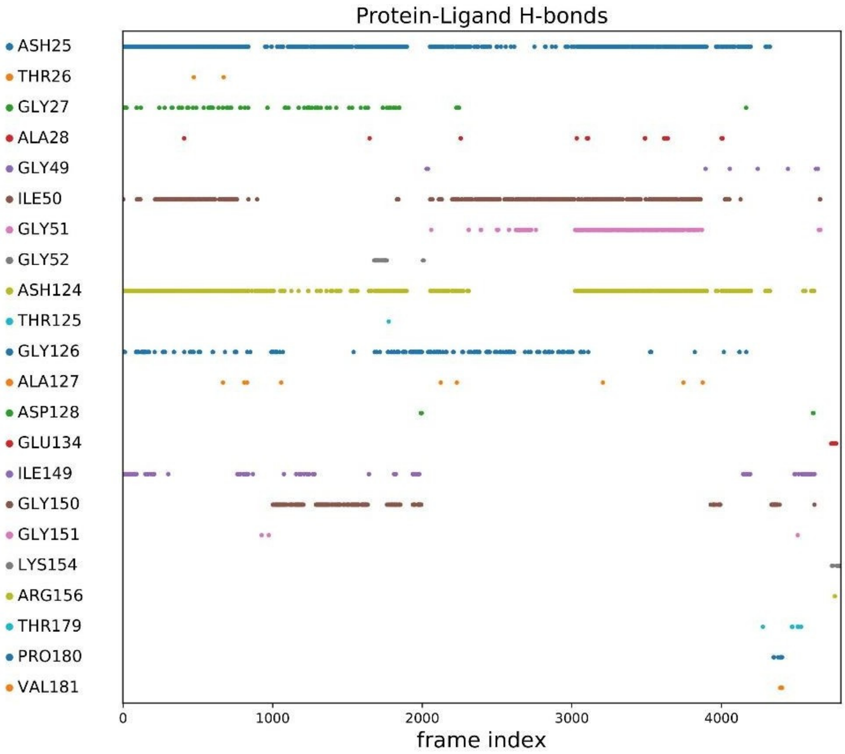

Figure S3. Hydrogen bond plot for xk263-HIVp under Pathway A dissociation. Time interval between each frame is 0.1 ns.

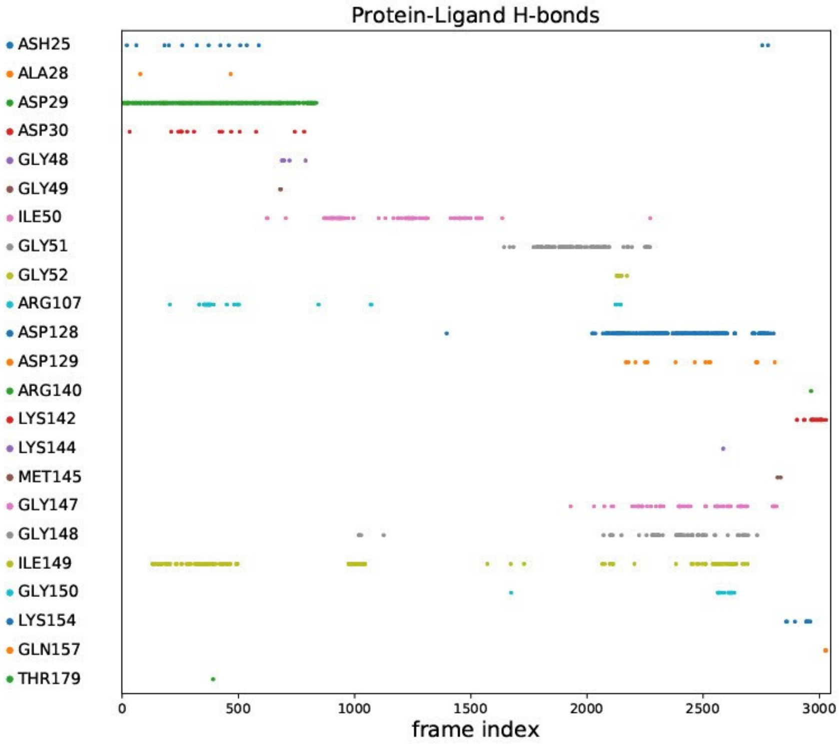

Figure S4. Hydrogen bond plot for ritonavir-HIVp under Pathway B dissociation. Time interval between each frame is 0.1 ns.

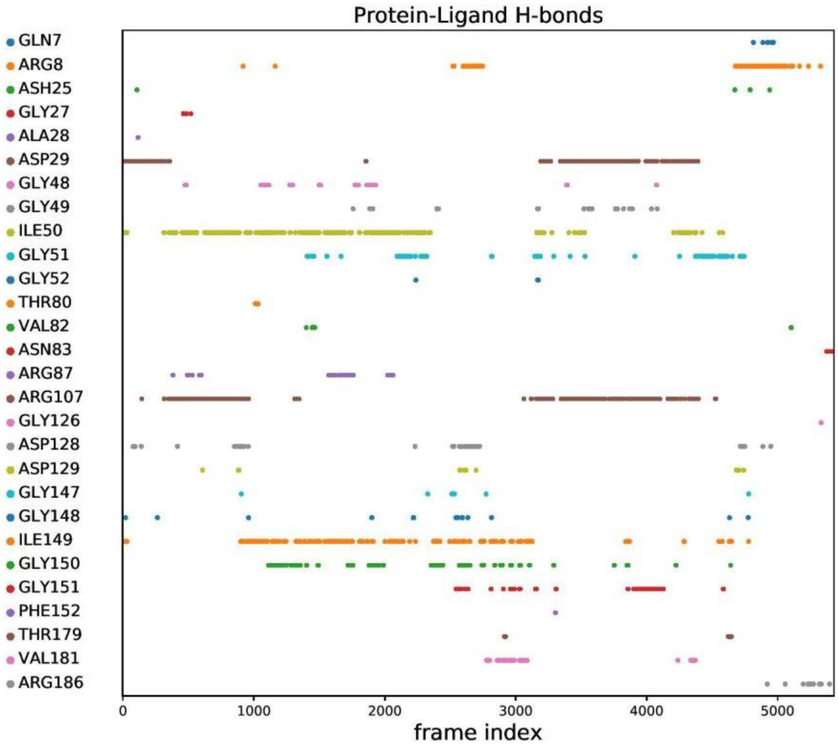

Figure S5. Hydrogen bond plot for ritonavir-HIVp under Pathway C dissociation. Time interval between each frame is 0.1 ns.

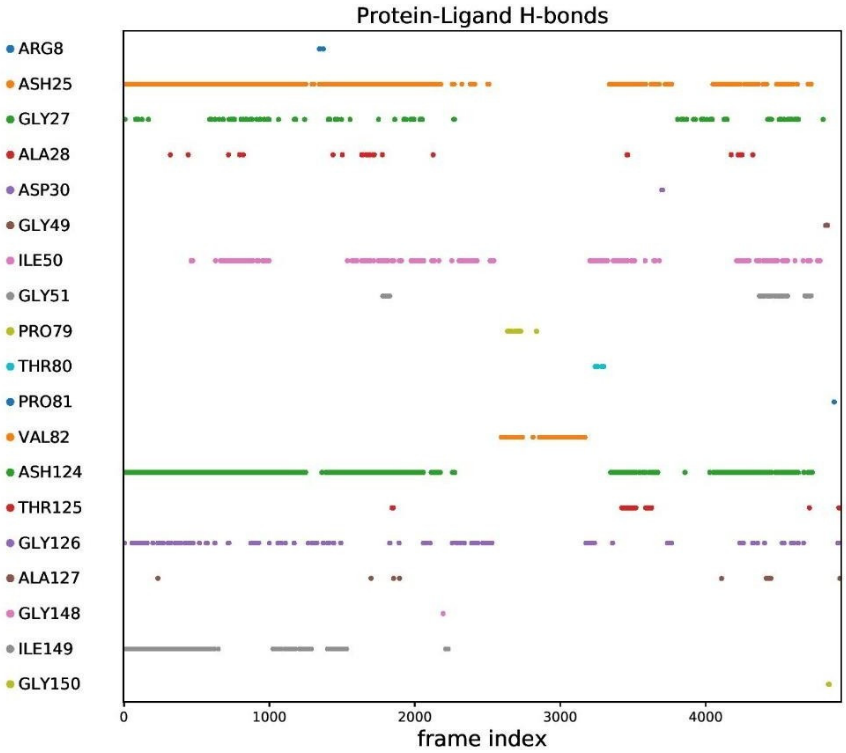

Figure S6. Hydrogen bond plot for xk263-HIVp under Pathway C dissociation. Time interval between each frame is 0.1 ns.

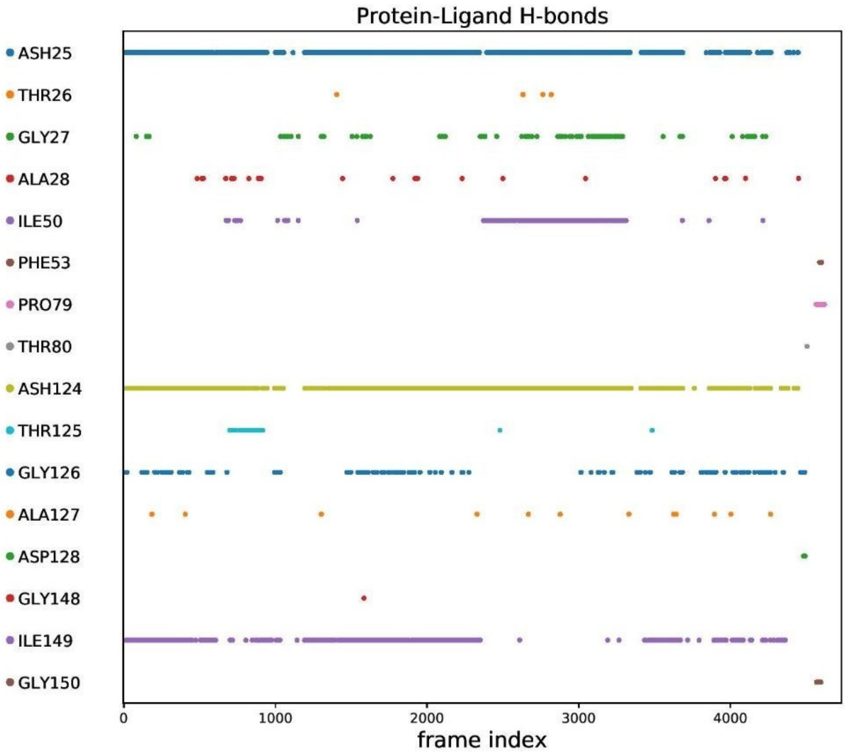

Figure S7. Hydrogen bond plot for xk263-HIVp dissociation with closed flaps. Time interval between each frame is 0.1 ns.

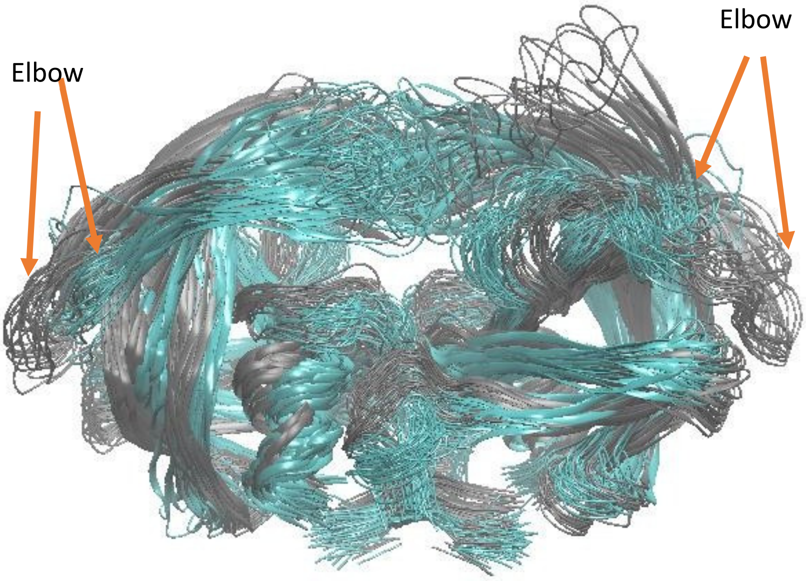

Figure S8. HIVp conformations in ritonavir dissociation under Pathway A (grey) and ritonavir Association 2 (cyan). Conformations were taken everything 10 ns. Elbow region is more twisted in association trajectory, which resulted in no overlapped HIVp conformation between dissociation and association.

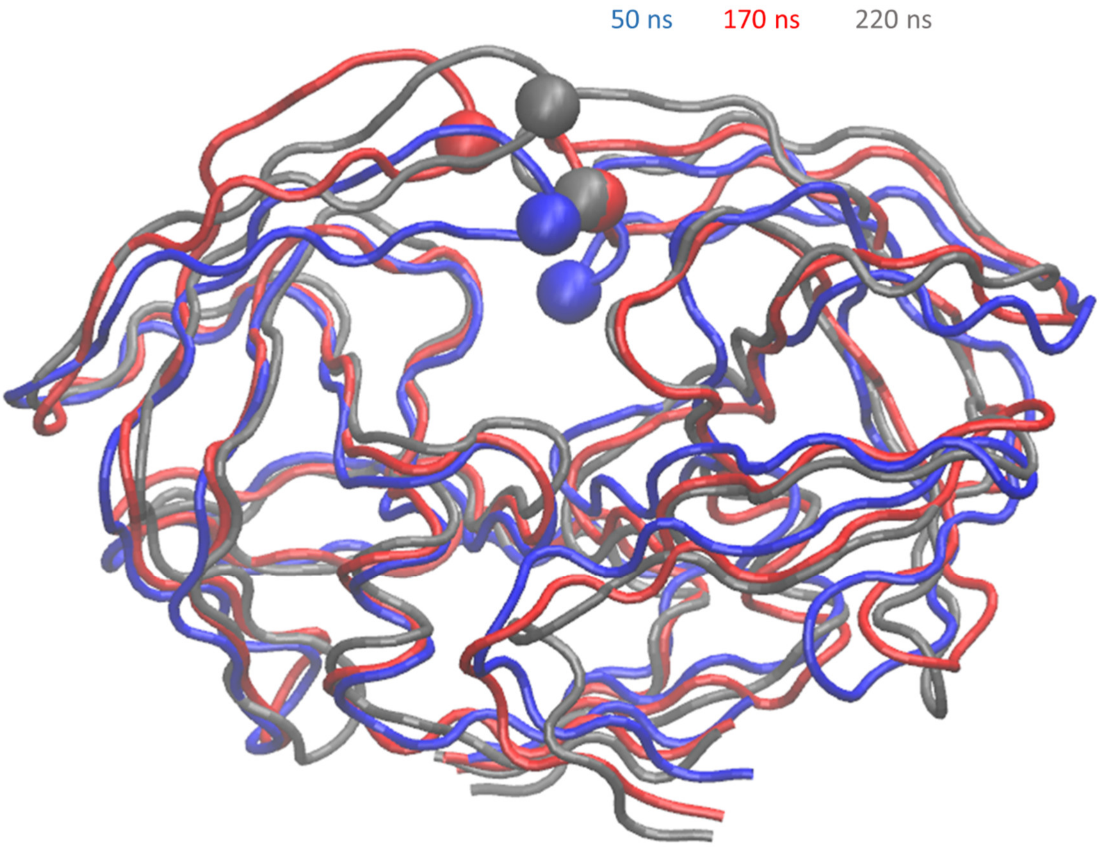

Figure S9. HIVp conformations that found many overlapping frames in ritonavir association. Snapshots are taken at 50 ns (blue), 170 ns (red) and 220 ns (grey) during ritonavir dissociation under pathway A. Alpha-Carbon of Ile 50/149 are shown in VDW representation.

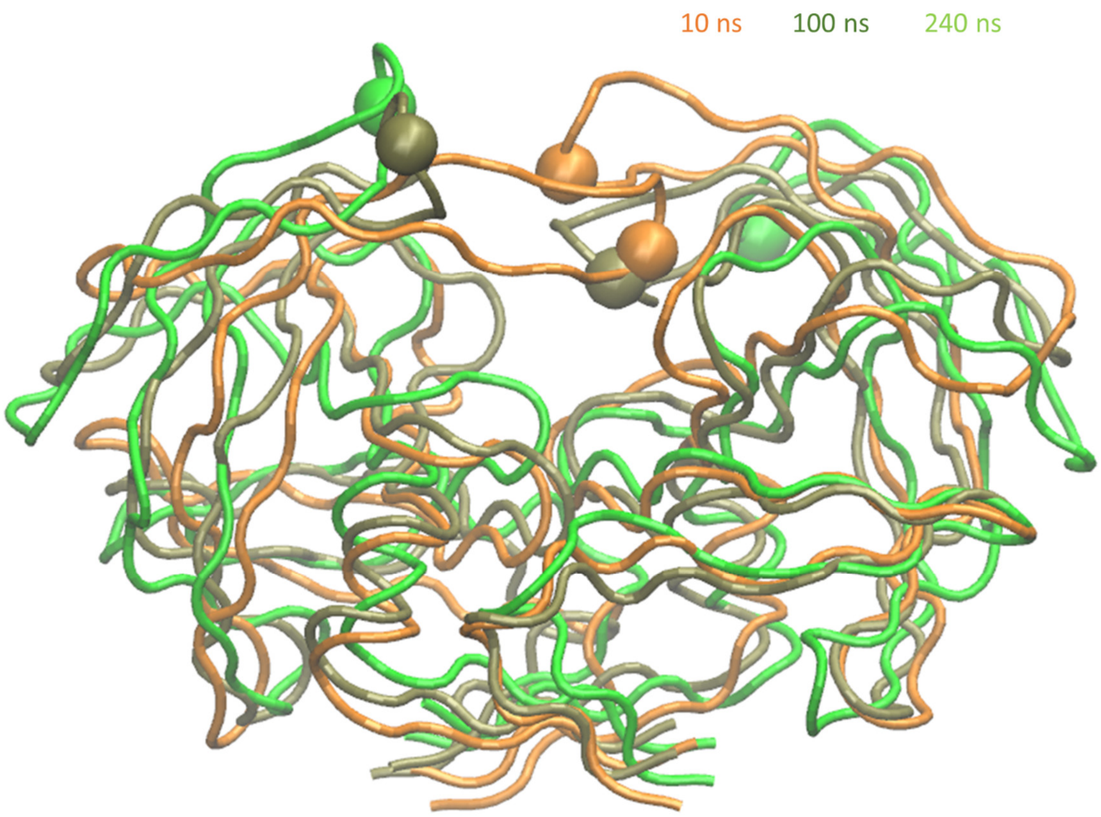

Figure S10. HIVp conformations that found no overlapping frames in any of ritonavir association trajectories. Snapshots are taken at 10 ns (orange), 100 ns (tan) and 240 ns (green) during ritonavir dissociation under pathway A. Alpha-Carbon of Ile 50/149 are shown in VDW representation.

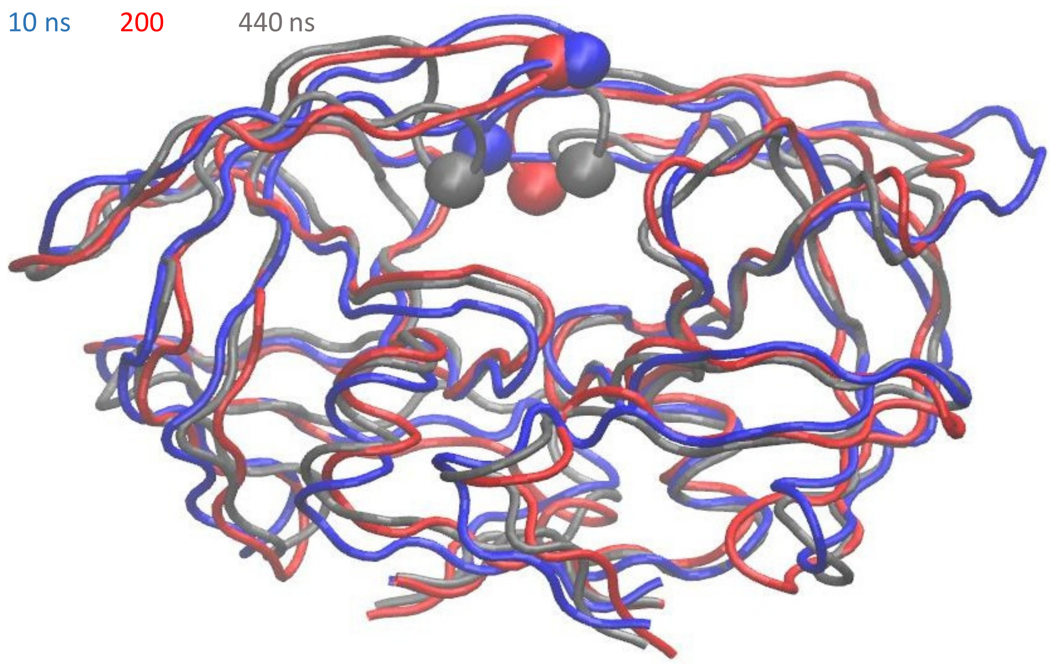

Figure S11. HIVp conformations that found many overlapping frames in xk263 association. Snapshots are taken at 10 ns (blue), 200 ns (red) and 440 ns (grey) during xk263 dissociation under pathway A. Alpha-Carbon of Ile 50/149 are shown in VDW representation.

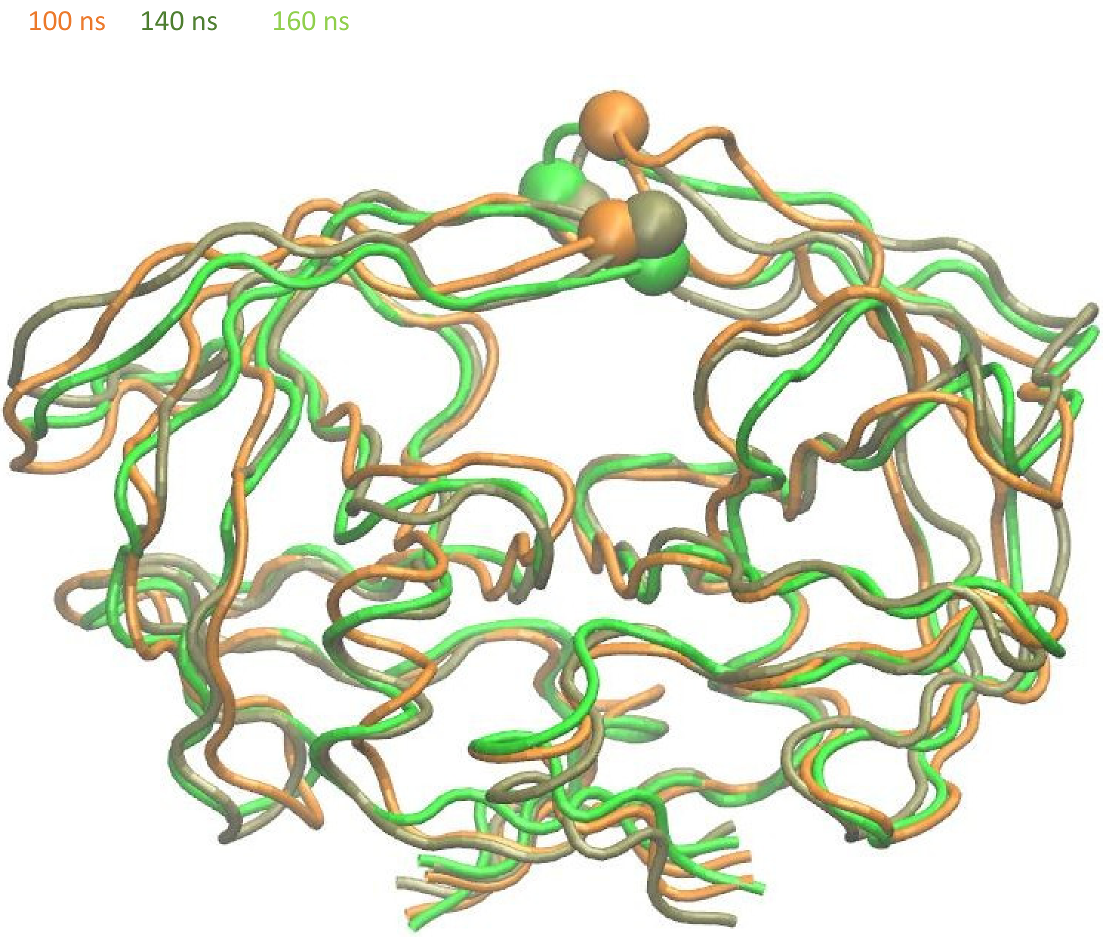

Figure S12. HIVp conformations that found no overlapping frames in any of xk263 association trajectories. Snapshots are taken at 100 ns (orange), 140 ns (tan) and 160 ns (green) during xk263 dissociation under pathway A. Alpha-Carbon of Ile 50/149 are shown in VDW representation.

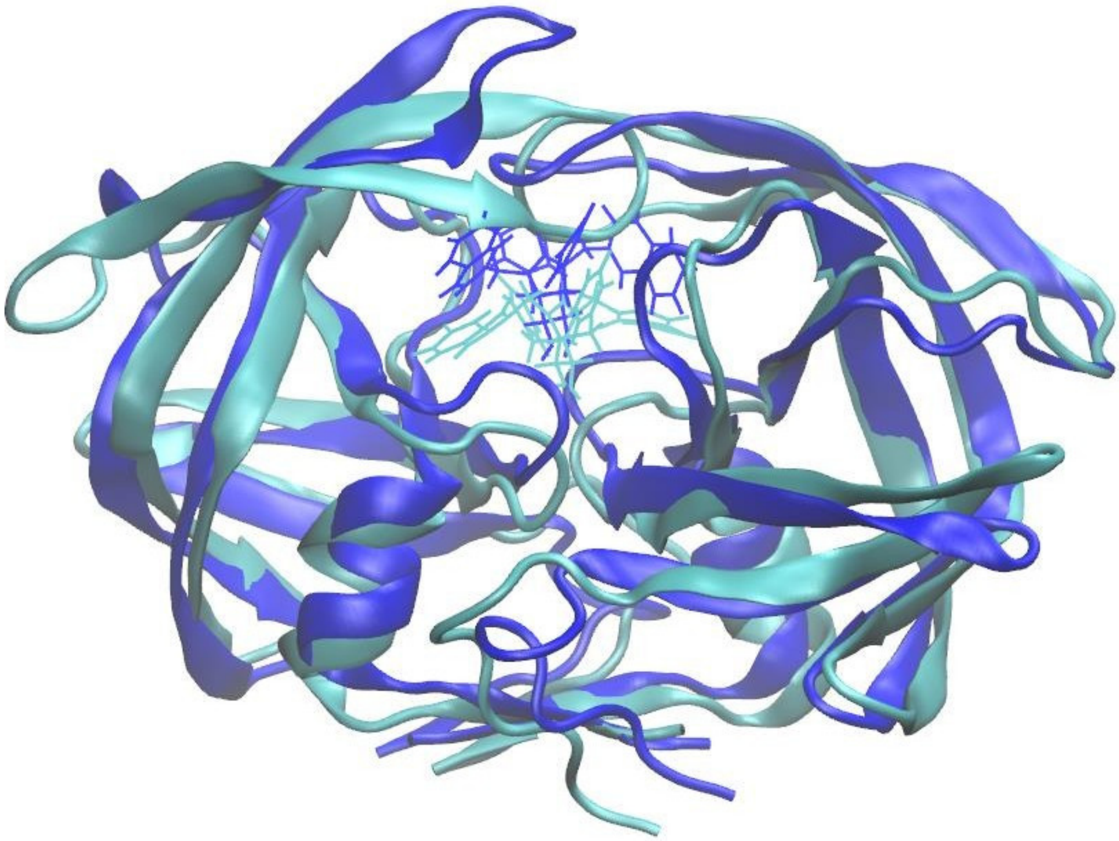

Figure S13. Observed flap stacking in xk263 dissociation. Snapshots of xk263-HIVp are taken at 0 ns (cyan) and 106.8 ns (blue). Xk263 is shown in line.
